# Influence of micro and nano particles on the mechanical, morphological and thermal properties of HDPE/PP blends

**DOI:** 10.1038/s41598-026-60009-8

**Published:** 2026-07-03

**Authors:** A. Koriem, A. M. Ollick, M. Elhadary, Ibrahim M. El-Sherbiny, A. Gomma

**Affiliations:** 1 Department of Mechanical Engineering, Faculty of Engineering, Alexandria University, Alexandria, Egypt; 2 Center for Materials Science, Zewail City of Science and Technology, Giza, Egypt

**Keywords:** Polymer blend, Nanocomposites, Mechanical properties, Morphology, Chemistry, Engineering, Materials science, Nanoscience and technology

## Abstract

Improving the mechanical performance of high-density polyethylene (HDPE) and polypropylene (PP) blends remains a significant challenge in materials science. Due to their inherent immiscibility and weak interfacial adhesion, these blends require suitable additives to enhance interfacial interaction and improve mechanical properties for practical applications. In this study, a series of HDPE/PP blends with HDPE contents of 25, 50, and 75 wt%, in addition to neat HDPE and PP were examined to determine the optimal base composition. The blend containing 75% HDPE was selected as the optimal composition due to its balanced mechanical performance. This blend was subsequently modified with silica, carbon black, Ethylene propylene diene monomer (EPDM), and their hybrid combinations at 5 wt% to evaluate their effectiveness in enhancing the overall performance of the material. In addition, fumed nano-silica was incorporated at 1, 3, and 5 wt% to examine its influence on the morphological, mechanical and thermal behavior of the blends. Mechanical performance was evaluated using tensile, hardness tests and impact tests, while phase morphology was investigated by scanning electron microscopy (SEM), and thermal behavior was evaluated by thermogravimetric analysis (TGA). The results demonstrate that micro-silica improved yield strength, elongation, and toughness by approximately 17%, 43%, and 72%, respectively, compared with the neat blend, while carbon black mainly enhanced yield strength by 19%. In contrast, EPDM led to an overall reduction in mechanical performance. Nano-silica produced the most significant enhancement, even at low concentrations. At only 1 wt%, nano-silica increased yield strength, elongation and toughness by nearly 11%, 98% and 120%, respectively, whereas the 3 wt% nano-silica formulation achieved the highest overall improvement, with yield strength, elongation and toughness enhanced by 13%, 120% and 150%, respectively. Thermogravimetric analysis revealed that the thermal stability of HDPE/PP blends influenced by both composition and additive type. Nano-silica exhibited the most pronounced stabilizing effect compared to micro-additives. These findings demonstrate that nano-silica is a highly efficient modifier for HDPE/PP blends and provides a practical route for producing high-performance and functional polyolefin materials for engineering and industrial applications.

## Introduction

Growing awareness of global warming, environmental sustainability, and waste management has intensified interest in plastic waste recycling. Due to the extensive use of polymers, particularly polyolefins, the treatment and recycling of solid plastic waste have become major environmental challenges. Among these polymers, polyethylene (PE) and polypropylene (PP) constitute a large portion of the total plastic waste, making them two of the most abundant materials in municipal solid waste streams^1–3^. In real recycling processes, complete separation of polyolefins is often economically and technically unfeasible by common separation techniques such as flotation because of the similar densities of the polymers, leading to mixed PE/PP waste with inferior properties. Consequently, recycling them together as a blend represents a promising approach to reduce plastic waste and mitigate environmental impact^4–7^.

Several studies^8–13^ have examined PE/PP blends, including systems containing high-density polyethylene (HDPE), low-density polyethylene (LDPE), and linear low-density polyethylene (LLDPE). However, these blends are generally immiscible and incompatible because of poor interfacial adhesion between the components, which limits their mechanical performance and, their applications. Therefore, the incorporation of suitable additives is essential to enhance interfacial interaction, improve phase dispersion, and promote more effective load transfer across the blend components. Morphological and thermal analyses have shown that PE/PP blends exhibit two distinct melting peaks in differential scanning calorimetry (DSC), droplets of the minor phase dispersed within the matrix as observed by scanning electron microscopy (SEM), and characteristic crystalline peaks in X-ray diffraction (XRD)^14–16^. PE and PP account for approximately 70% of total plastic solid waste, with PE/PP ratios ranging from 80:20 to 65:35 depending on geographical region^17^. This underscores the relevance of studying PE/PP blends to develop more efficient recycling and material reuse strategies.

Shan et al.^18^ demonstrated that the mechanical properties of LDPE/PP blends are highly composition-dependent. Strength and modulus increased with rising PP content, while elongation and impact strength exhibited non-linear trends, with maximum impact strength observed at 20% PP in LDPE/PP blends. Rosales et al.^19^ have reported that virgin and recycled LDPE/PP blends exhibit comparable mechanical behavior. This indicates that testing and analyzing newly prepared materials can provide meaningful insight into the expected performance of recycled blends, at least as an initial evaluation, despite possible variations in composition and the presence of impurities.

Siraj et al.^20^ indicated that incorporating silica microparticles into HDPE can influence the mechanical performance and density of the resulting composites. Silica with specific particle sizes 25 μm, have demonstrated improvements in toughness and stiffness. These findings highlight the potential of silica, particularly locally sourced sand, as a low-cost filler for lightweight polymer composites. Dil and Favis^21^ demonstrated that the scale of silica particles strongly affects their behavior in polylactic acid (PLA) and LDPE blends. Micro-sized silica shows limited mobility and tends to remain in the phase with lower interfacial tension, while nano-silica can migrate more easily, with individual nanoparticles reaching their thermodynamic equilibrium locations. Their findings highlight how particle size governs dispersion, migration, and final localization in immiscible polymer blends.

Li et al.^22^ studied High-density polyethylene (HDPE) composites containing propylene-ethylene random copolymers (PEC) and carbon black (CB). They showed that carbon black selectively locates in the HDPE phase and forms a percolated network at around 5 wt%, significantly influencing the blend morphology. Increasing CB content enhances stiffness, yield strength, and electrical conductivity, while reducing the ductility and impact strength of the composite.

Penava et al.^23^ further reported that incorporating EPDM as a compatibilizer in PP/LDPE blends significantly improved interfacial adhesion and mechanical properties, particularly enhancing impact strength in LDPE-rich compositions, as confirmed by mechanical and SEM analyses. Similarly, Dubey et al.^24^ investigated the influence of EPDM at high loading ratios up to 50 wt% on CB-filled PP/PE blends and found that while EPDM improved elongation at break and compatibility in unfilled blends, its effect was limited in CB-filled systems.

Zou et al.^25^ Studied immiscible polymer blends and have shown that fumed silica nanoparticles can significantly alter blend morphology and rheology. These particles reduce droplet size, promote droplet clustering, and can even form cluster structures regardless of whether they are wetted by the continuous or dispersed phase. Many researchers^26–31^ reported that incorporating silica nanoparticles into immiscible blends enhanced tensile strength, Young’s modulus, and impact strength owing to the reinforcing effect of nanoparticles and their high surface area. Nevertheless, excessive nanoparticle loading was found to decrease elongation at break and impact strength due to agglomeration and reduced matrix flexibility.

Previous studies have highlighted the importance of filler incorporation and morphology control in improving the performance of immiscible polymer blends. Maou et al. investigated the effect of chemically modified date palm fibers on Polyvinyl chloride (PVC) and HDPE composites and reported significant improvements in thermo-physical properties due to enhanced fiber–matrix interactions^32^. In another study, compatibilization using maleic anhydride was shown to improve the compatibility of immiscible PVC/HDPE blends, resulting in improved thermal and mechanical performance^33^. Furthermore, the incorporation of recycled polyethylene, calcium carbonate, and metal stearates was found to improve the thermo-mechanical behavior and thermal stability of PVC-based systems^34^. Recent morphological investigations of PVC/PE blends have also demonstrated the critical role of compatibilizers and phase morphology in achieving improved dispersion, interfacial adhesion, and overall material performance^35^.

In recent decades, numerous studies have shown that incorporating small amounts of inorganic nanoparticles lower than 5 wt%^36–39^, into polymer matrices can significantly enhance various properties, including mechanical, thermal performance and gas barrier behavior. Due the growing scientific and industrial interest in PP/PE blends, it is essential to deepen the understanding of the relationship between morphology and mechanical properties to achieve optimized performance.

Despite their similar chemical structures, PE and PP exhibit distinct thermal degradation behaviors arising from differences in backbone structure and chain stability^15^. Polypropylene, containing tertiary carbon atoms, is generally more susceptible to chain scission reactions than polyethylene. When blended, the degradation behavior of PE/PP systems does not always follow simple rules, and interactions between polymer phases may influence degradation onset, kinetics, and volatile evolution. Therefore, understanding the thermal stability of PE/PP blends is essential for optimizing the properties and improving material performance^40–43^.

Thermogravimetric analysis (TGA) is a powerful and widely applied technique for evaluating polymer thermal stability, degradation kinetics, and residue formation. Parameters such as the temperatures corresponding to 5% and 10% mass loss (T₅ and T₁₀) and the temperature at maximum degradation rate (T_max_) provide valuable insight into degradation mechanisms and stability trends. For polyolefins, which typically undergo single-step volatilization with minimal char formation, subtle shifts in these characteristic temperatures can reveal important compositional and interfacial effects^42,44^.

The selected blend ratios (25, 50, and 75 wt% HDPE) were chosen in this study to represent PP-rich, intermediate, and PE-rich compositions and to identify the optimum blend composition prior to additive incorporation. A PE-rich composition (75% HDPE) was selected based on both performance and practical considerations. This composition provides a balanced combination of strength and ductility compared to other blend ratios, making it a suitable model system for evaluating the influence of different additives. In addition, previous studies have shown that PE-rich PE/PP blends exhibit improved overall mechanical behavior^45^, supporting the selection of this composition. From an industrial perspective, such compositions are also representative of typical ratios encountered in mixed polyolefin waste streams^17^, where complete separation is rarely achieved and PE-rich fractions are commonly obtained.

Previous studies have mainly focused on the effects of individual additives on polymer blends, while comparisons among different additives such as silica, CB, EPDM and their hybrid combinations remain limited^2,22,23^. The novelty of this study lies in providing a comprehensive evaluation of multiple additives and their hybrids processed and tested using the same experimental parameters, enabling a direct and reliable comparison of their impact on the mechanical properties of polymer blends. This study provides insight into the influence of filler type and particle size on the mechanical, thermal, and morphological characteristics of HDPE/PP blends. The direct comparison of these fillers highlights how different modification strategies influence phase morphology, interfacial interactions, and the resulting performance of HDPE/PP blends. The present work demonstrates that different modifier classes affect blend behavior differently, leading to distinct changes in toughness, ductility, thermal stability, and stress concentration characteristics. The findings contribute to understanding the structure–property relationships governing blend morphology and mechanical performance. By incorporating these additives into the optimized blend, this work provides a practical route for producing high-performance HDPE/PP composites with significantly improved mechanical strength, toughness, and functional performance.

## Experimental

### Materials

#### Base polymers

Polymer blends were prepared based on commercial high-density polyethylene (HDPE) 6070UA with melt flow index of 7.5 g /10 min and density of 960 Kg/m^3^, provided by SIDPEC petrochemicals company – Egypt, and polypropylene block copolymer (PP) BD265MO with melt flow index 7 g /10 min and density 910 kg/m^3^, provided by Borouge petrochemicals company – United Arab Emirates.

#### Additives

Silica (Mansil 175G) was supplied by Akrochem Corporation – USA, average particle size 18–20 μm, specific surface area 170 m^2^/g, and bulk density 330 g/liter. Carbon black N550 was supplied by Macrochem - Poland, specific surface area 40 m^2^/g, pH value 7, and bulk density 290–370 g/liter. Ethylene propylene diene monomer (EPDM), NORDEL 4725P was supplied by Dupont Dow Elastomers – USA, containing ethylene 69–71 wt%, propylene 24–26 wt% and ethylene norbornene 4-5wt.%. Moony viscosity of EPDM is 25 at 125 °C. Fumed silica nanoparticles, FUSIL 200 was kindly supplied by Dalian Fuchang chemical company- China, average particle size 5–40 nm, specific surface area 200 m^2^/g, and bulk density 30–60 g/liter.

### Blend preparation

Blends of HDPE and PP, with and without additives, were initially mixed in a laboratory mixer for 15 min to achieve a homogeneous dispersion of all components. The resulting mixture was then fed directly into a two-roll mill (HAPRO-KL157). The mill was operated at 200 °C with a roll speed of approximately 20 rpm, and the average compounding time was 10 min^45,46^. The material was repeatedly folded and passed through the rolls during milling to improve the mixing and additive dispersion. Subsequently, the blended materials were compression molded using a platen press (HAPRO-PL300) for 10 min at 200 °C under a pressure of 95 bar. Plaques of 210 mm length, 210 mm width and 3 mm thickness were obtained. A detailed description of the materials examined in this work is provided in Table 1.

**Table 1:** Samples composition.

Sample	PE (wt%)	PP (wt%)	Silica (wt%)	CB (wt%)	EPDM (wt%)	Nano Silica (wt%)
PE	100	0	0	0	0	0
PE75	75	25	0	0	0	0
PE50	50	50	0	0	0	0
PE25	25	75	0	0	0	0
PP	0	100	0	0	0	0
PE75/Si	71.25	23.75	5	0	0	0
PE75/CB	71.25	23.75	0	5	0	0
PE75/EPDM	71.25	23.75	0	0	5	0
PE75/Si/CB	71.25	23.75	2.5	2.5	0	0
PE75/Si/EPDM	71.25	23.75	2.5	0	2.5	0
PE75/CB/EPDM	71.25	23.75	0	2.5	2.5	0
PE75/N1	74.25	24.75	0	0	0	1
PE75/N3	72.75	24.25	0	0	0	3
PE75/N5	71.25	23.75	0	0	0	5

### Tensile test

Tensile properties were evaluated using a universal testing machine (Mecmesin MultiTest 5Xt) equipped with a 5 kN load cell. Standard ASTM D638 Type I dumbbell-shaped specimens were prepared from the compression-molded sheets using a standard sample cutter, and tensile testing was carried out in accordance with ASTM D638^47^ at a crosshead speed of 50 mm/min, under controlled laboratory conditions (temperature 23 ± 2 °C, relative humidity 50 ± 5%). Modulus of elasticity was calculated as the slope of stress-strain curve in the elastic region. Toughness was calculated as the total area under stress-strain curve. For each material composition, three specimens were tested^48,49^, and the average values of the measured tensile parameters were reported with standard deviations.

### Hardness test

Hardness measurements were performed using a Shore D durometer in accordance with the recommendations of ASTM D2240^50^. All tests were conducted at room temperature under atmospheric conditions. For each composition, at least three specimens were tested, and a minimum of five measurements were recorded on each specimen. The reported hardness values represent the average of fifteen measurements for each composition.

### Impact test

Charpy impact tests were performed according to ISO 179 using an Instron Ceast 9050 impact tester (Italy), at an impact velocity of 3.5 m/s. Unnotched specimens with dimensions of 80 × 10 × 3 mm³ were tested at room temperature (23 ± 2 °C). The absorbed energy was recorded, and the impact strength (kJ/m²) was calculated based on the cross-sectional area. Impact tests were performed on selected representative compositions to highlight the effect of nano-silica loading on impact strength. For each composition, at least five specimens were tested, and average values with standard deviation were reported.

### Morphology

To examine the developed microstructure, the specimens were first notched, immersed in liquid nitrogen for 15 min, and then cryo-fractured to ensure a brittle fracture surface, preventing plastic deformation and revealing the true internal morphology^13^. The fractured surfaces were then coated with a thin layer of gold to improve the image’s conductivity and quality and imaged using a JEOL JCM-7000 scanning electron microscope (SEM).

### Thermogravimetric analysis (TGA)

Thermogravimetric analysis (TGA) was carried out using a THASS TGA i1000 thermogravimetric analyzer. The instrument measures mass change as a function of temperature under a controlled atmosphere and programmed heating conditions. Approximately 10–12 mg of each sample was placed in a standard titanium crucible and analyzed from 20 °C to 650 °C at a constant heating rate of 10 °C/min. All measurements were conducted under a nitrogen atmosphere to prevent oxidative degradation, with a purge gas flow rate of 20 mL/min. The characteristic degradation temperatures, including the temperatures at 5% and 10% mass loss (T₅ and T₁₀), the temperature corresponding to the maximum degradation rate (T_max_), and the residual mass at 650 °C, were determined from the TGA and derivative thermogravimetric (DTG) curves. All experiments were performed under identical conditions to ensure reliable comparison among neat blends and additive-modified compositions.

## Results and discussion

### Mechanical characteristics

#### Neat polymer blends

**Fig. 1:**
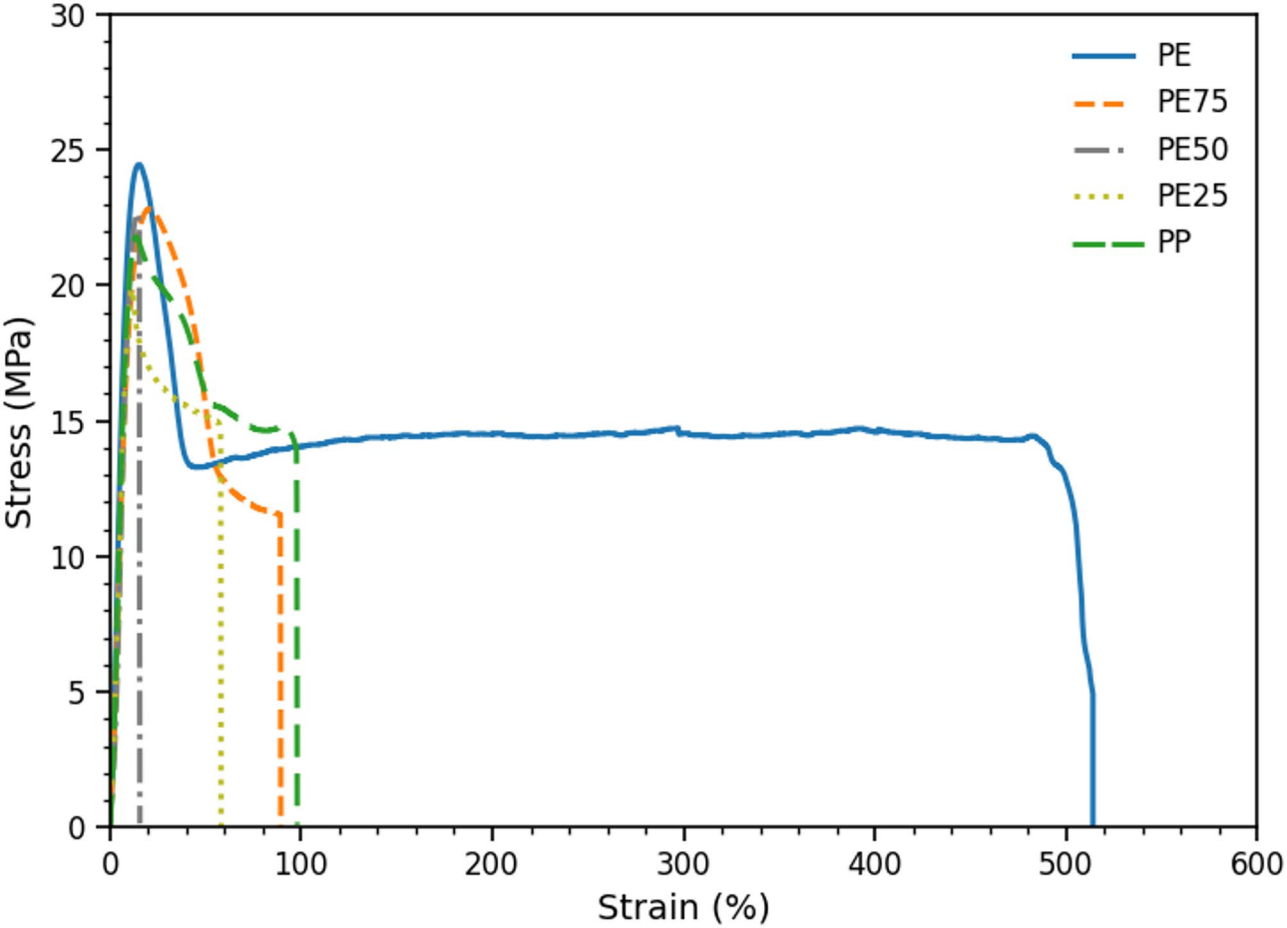
Stress-strain curves for PE, PP and neat blends (PE75, PE50, PE25).

The tensile behavior of the neat polymers highlights the contrast between the mechanical properties of PE and PP. Figure 1 shows the stress-strain curves for PE, PP and blends at various ratios. Neat PE has the highest yield stress (24.4 MPa), the greatest ductility (514% elongation), and toughness (75.4 MJ/m^3^). In contrast, it exhibits the lowest Shore D hardness (61.1), reflecting its soft and highly ductile nature. Stress–strain curve of PE displays a well-defined yield point followed by an extended strain-hardening region, reflecting its ability to undergo large plastic deformation before failure. In comparison, neat PP has a slightly lower yield stress (21.9 MPa) but significantly lower ductility (94.4%) and toughness (15.2 MJ/m^3^), while presenting the highest Shore D hardness (67.5), consistent with its higher crystallinity and inherently rigid molecular structure compared with PE, confirming its relatively brittle nature^49^.

**Fig. 2:**
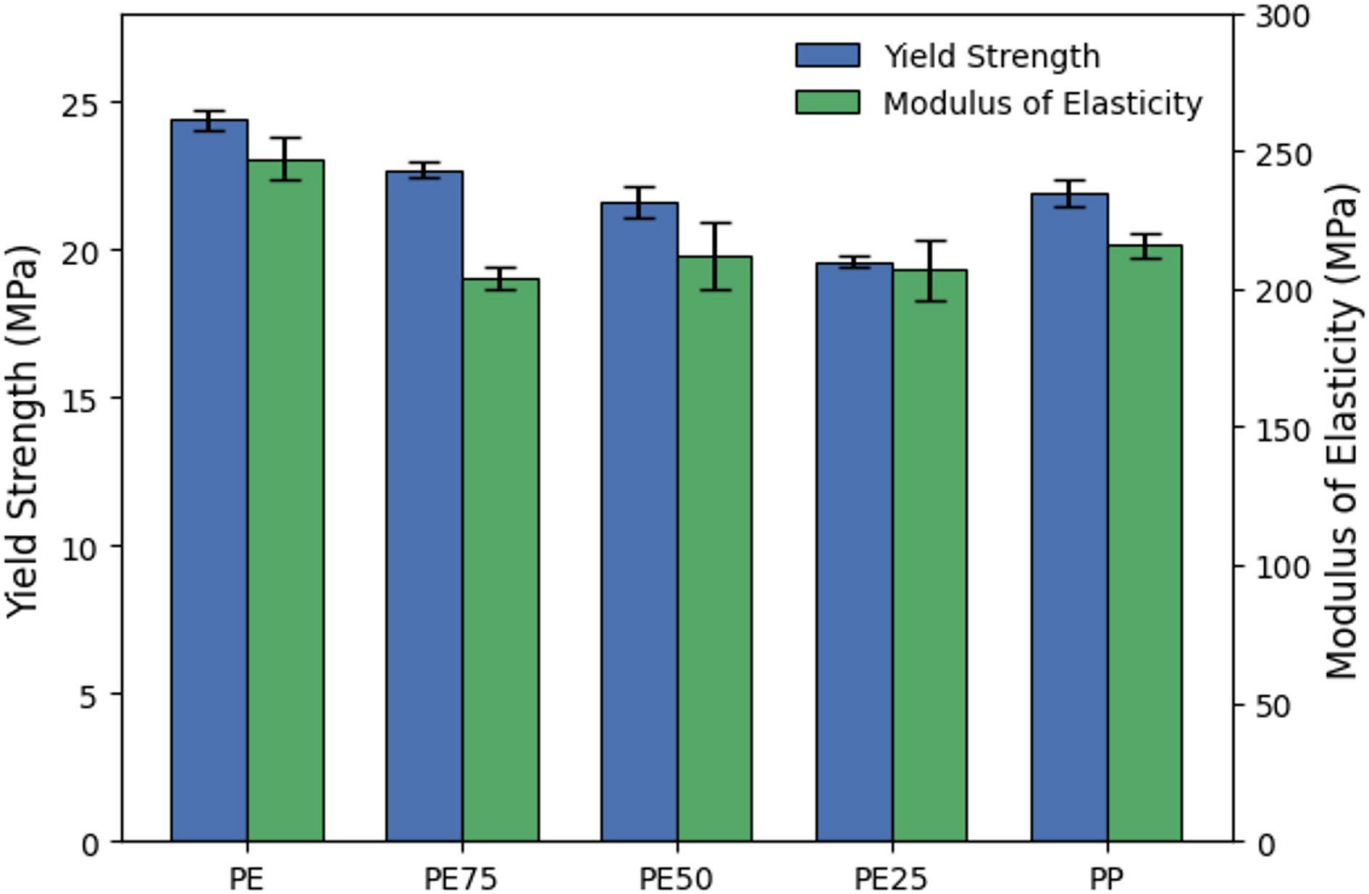
Yield stress and modulus of elasticity for PE, PP and neat blends (PE75, PE50, PE25).

**Fig. 3:**
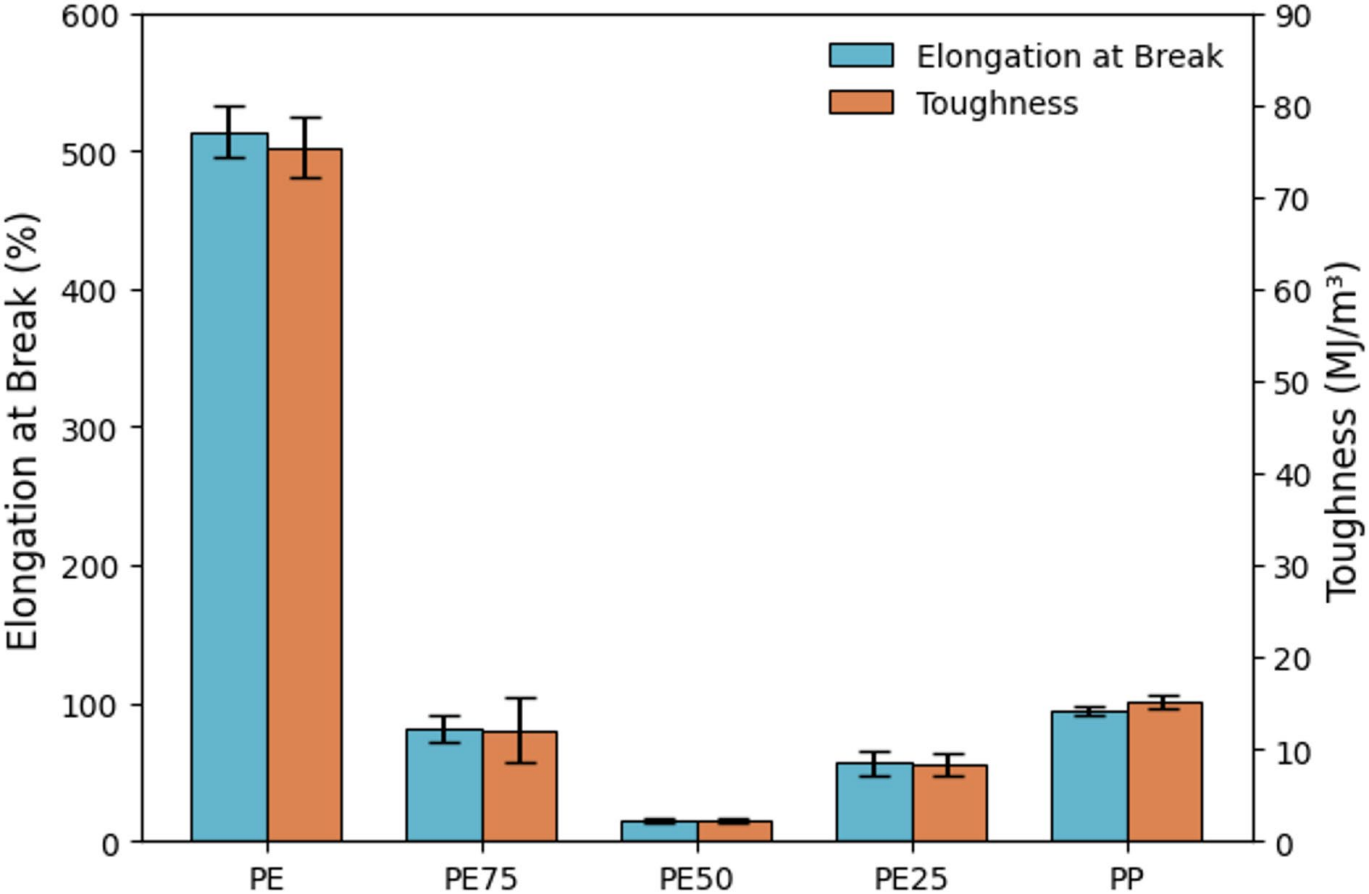
Elongation at break and Toughness for PE, PP and neat blends (PE75, PE50, PE25).

**Fig. 4:**
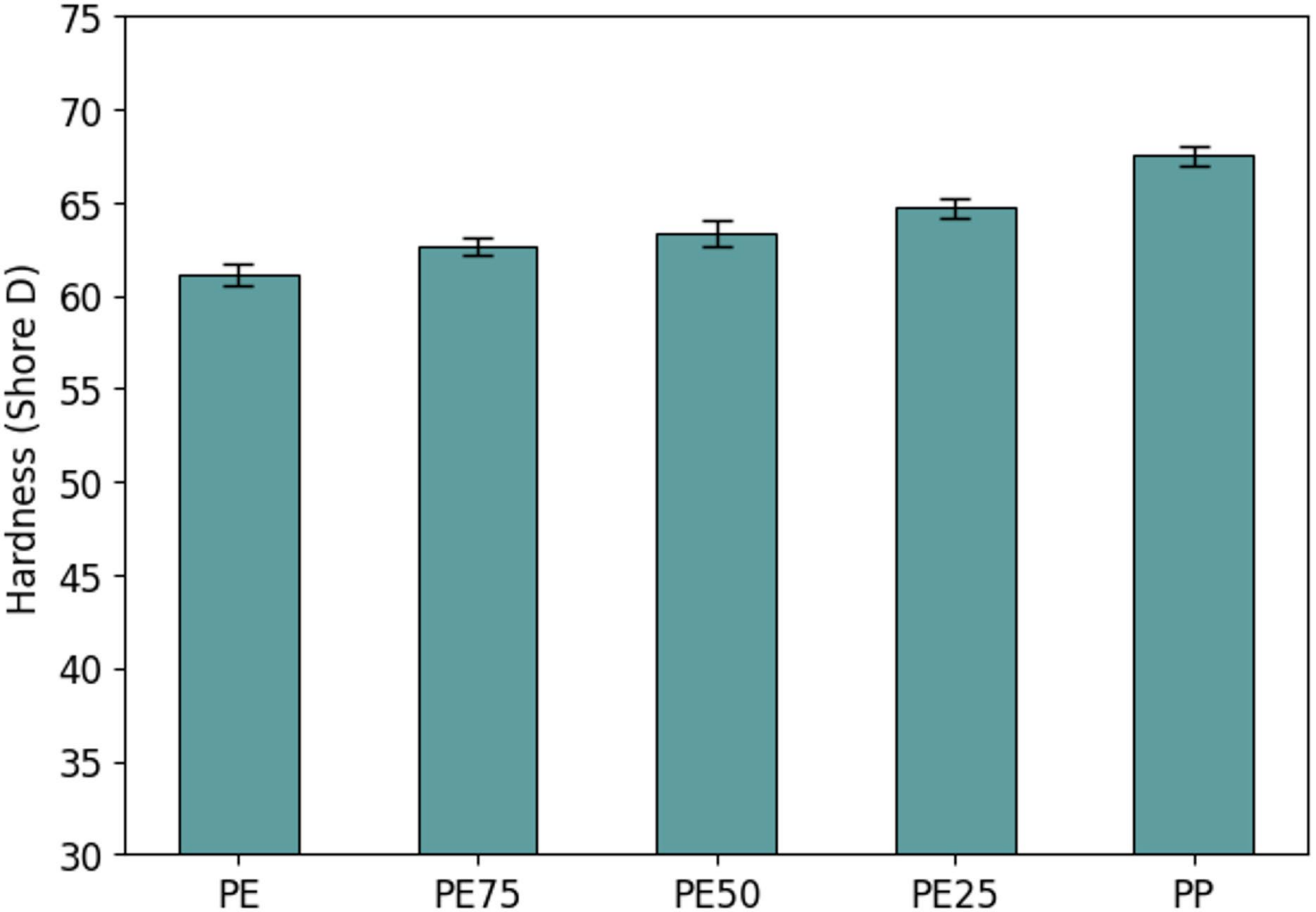
Hardness for PE, PP and neat blends (PE75, PE50, PE25).

Introducing PP into PE produces a clear and progressive reduction in ductility and toughness relative to neat PE, accompanied by a slight decrease in yield stress and an increase in hardness. Figure 2 shows yield stress and modulus of elasticity, for neat PE, PP and their blends. Figures 3 and 4 show the elongation at break, toughness and hardness, respectively. Table 2 presents the mechanical properties of neat PE, PP and their blends as mean values accompanied by standard deviations.

**Table 2:** Mechanical properties of neat polymer blends.

Property / composition	Yeild stress (Mpa)	Modulus of elastisity (Mpa)	Elongation at break (%)	Toughness (MJ/m^3^)	Hardness (Shore D)
PE	24.4 ± 0.34	247 ± 7.7	514 ± 18.4	75.4 ± 3.3	61.1 ± 0.6
PE75	22.7 ± 0.2	204 ± 4.3	81.9 ± 9.4	12.1 ± 3.5	62.6 ± 0.5
PE50	21.6 ± 0.55	212 ± 12	14.9 ± 1.3	2.2 ± 0.3	63.4 ± 0.7
PE25	19.6 ± 0.19	207 ± 11	56.5 ± 9.3	8.4 ± 1.2	64.7 ± 0.6
PP	21.9 ± 0.46	216 ± 4.5	94.4 ± 3.5	15.2 ± 0.7	67.5 ± 0.5

The PE75 blend shows a moderate yield stress of 22.7 MPa, positioned between PE and PP, but the elongation at break and toughness falls sharply to 81.9% and 12.1 MJ/m^3^, representing an approximate 84% reduction for both compared to neat PE, which indicates that PP has a much strong effect on post-yield deformation. The Shore D hardness increases slightly to 62.6 (2.5% increase) compared to neat PE, indicating that the introduction of PP contributes to increased rigidity^51^.

For PE50, the yield stress decreases further to 21.6 MPa, but the most dramatic change is the extremely low elongation at break (14.9%) and very low toughness (2.2 MJ/m^3^), representing a severe reduction of 97% for both properties compared to neat PE, showing a transition to a highly brittle behavior accompanied by a slight increase in hardness by 4%. This behavior has been reported by several authors^45,51^, who noted that PE50 exhibits the lowest ductility due to the co-continuous morphology formed between the two immiscible polymers.

Compared with neat PE, the PE25 blend exhibits a reduction in yield strength of approximately 20%, accompanied by a pronounced decrease in elongation at break of about 89% and toughness of nearly 89%. In contrast, the hardness increases by approximately 6%, indicating more stiffening effect as the PP phase becomes dominant in the blend morphology.

In general, the modulus of elasticity varies only slightly among all compositions (~ 204–212 MPa), indicating that their elastic behavior is not strongly dependent on composition. This is expected because both PE and PP possess similar crystalline structures that primarily control yielding and elasticity^12^. However, the ductility, strain hardening, and hardness is highly sensitive to blend composition ratio. As PP increases, the continuous ductile PE phase becomes increasingly disrupted, creating weak interfacial regions due to the poor compatibility between the two polymers. These weak interfaces act as stress concentrators and lead to premature failure, which explains the dramatic loss of elongation at break and toughness.

Among the investigated blends, the PE75 blend exhibits the best mechanical performance, while the PE50 blend represents the most brittle composition. These results highlight the inherent immiscibility of PE and PP and demonstrate the need for additives to restore toughness when blending these two polymers. As a result, the PE75 composition was selected for further modification using micro and nano fillers to enhance its mechanical performance.

#### Polymer blends with micro particles

**Fig. 5:**
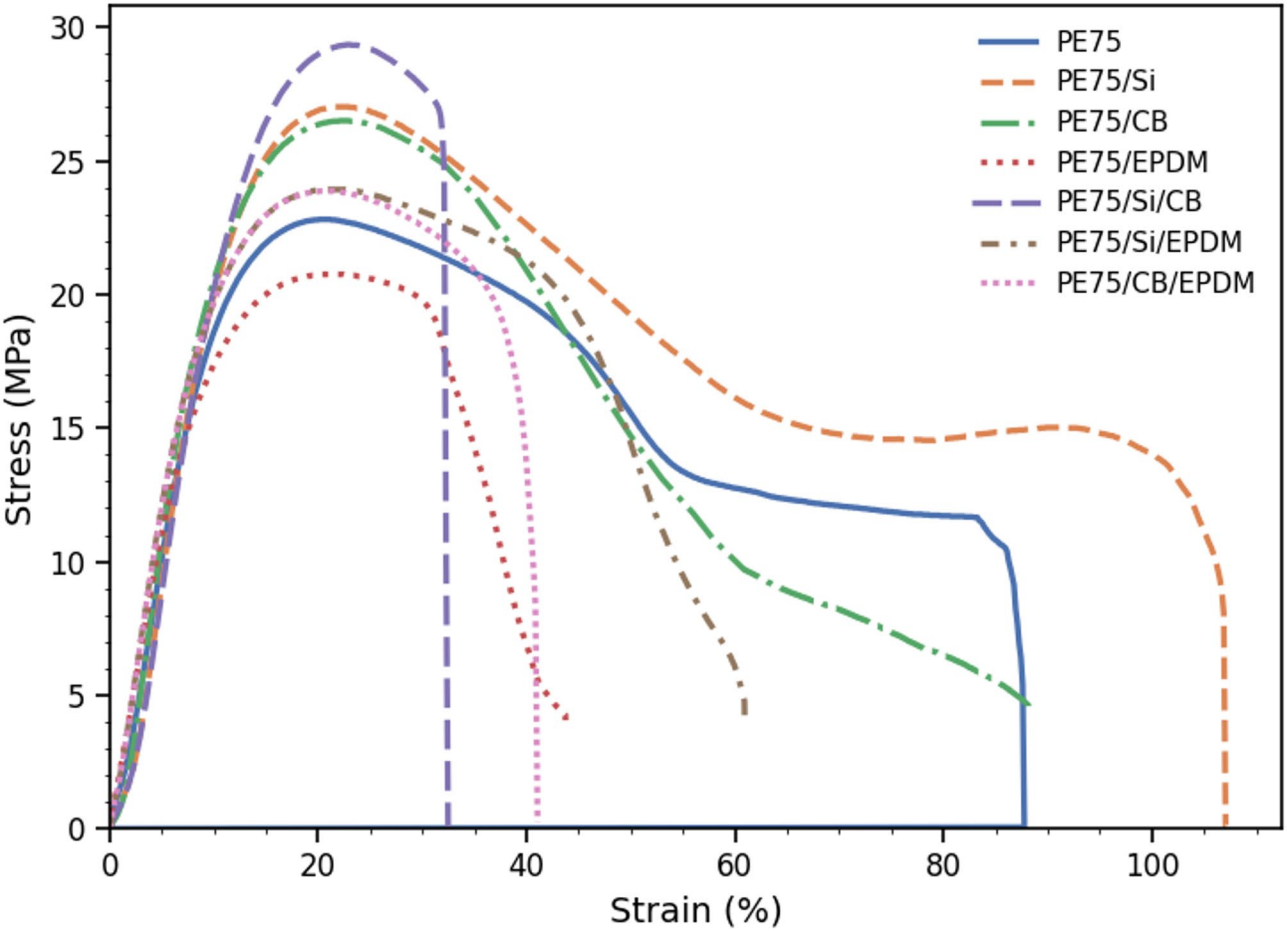
Stress-strain curves for neat PE75 blend and its micro-filled blends.

**Fig. 6:**
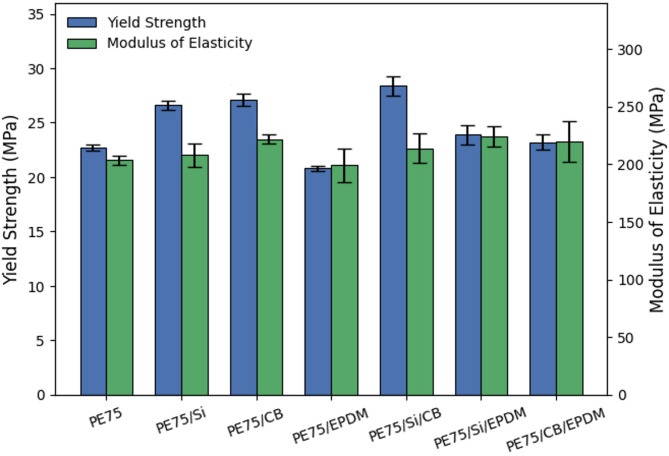
Yield stress and modulus of elasticity for neat PE75 blend and its micro-filled blends.

**Fig. 7:**
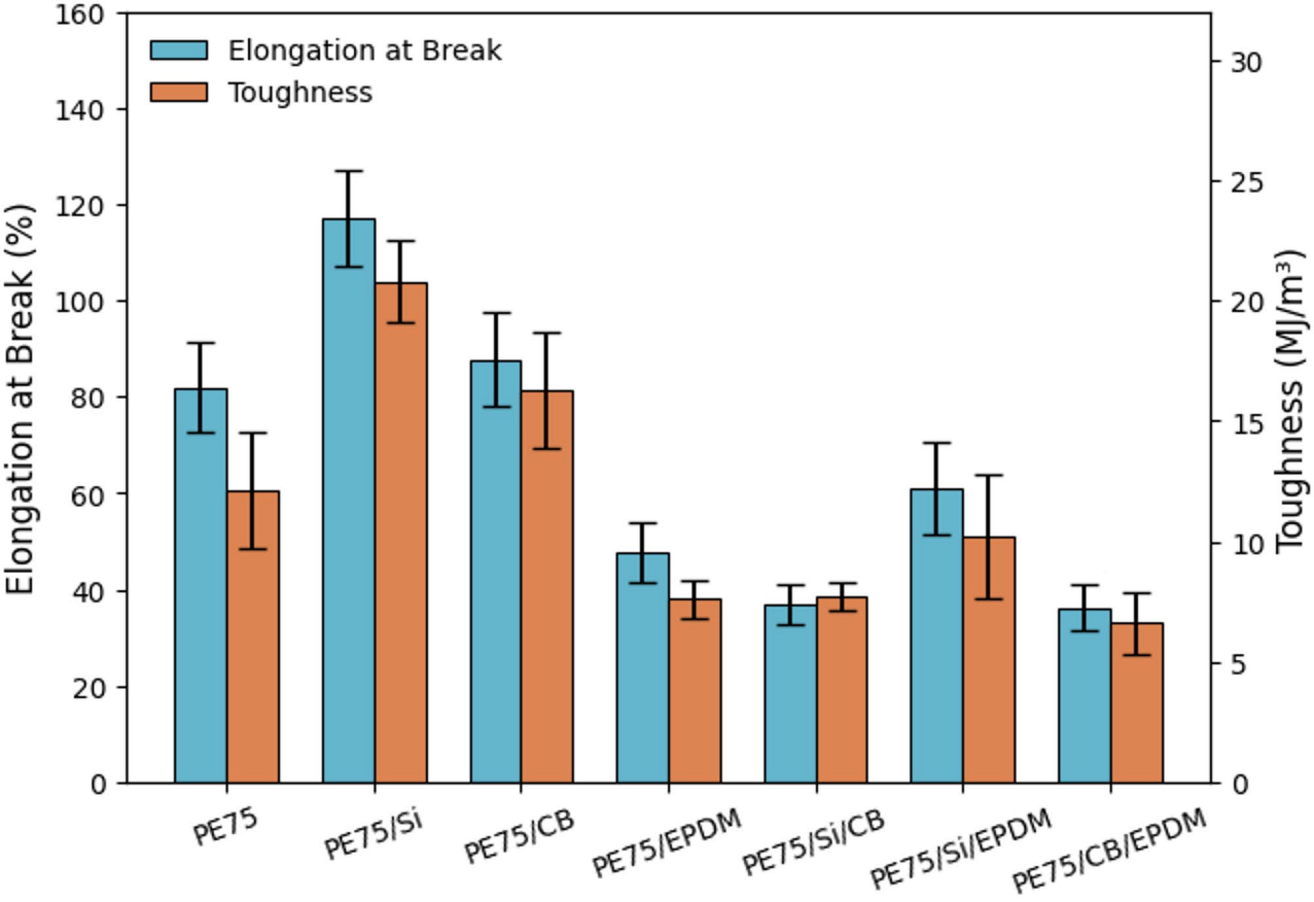
Elongation at break and toughness for neat PE75 blend and its micro-filled blends.

**Fig. 8:**
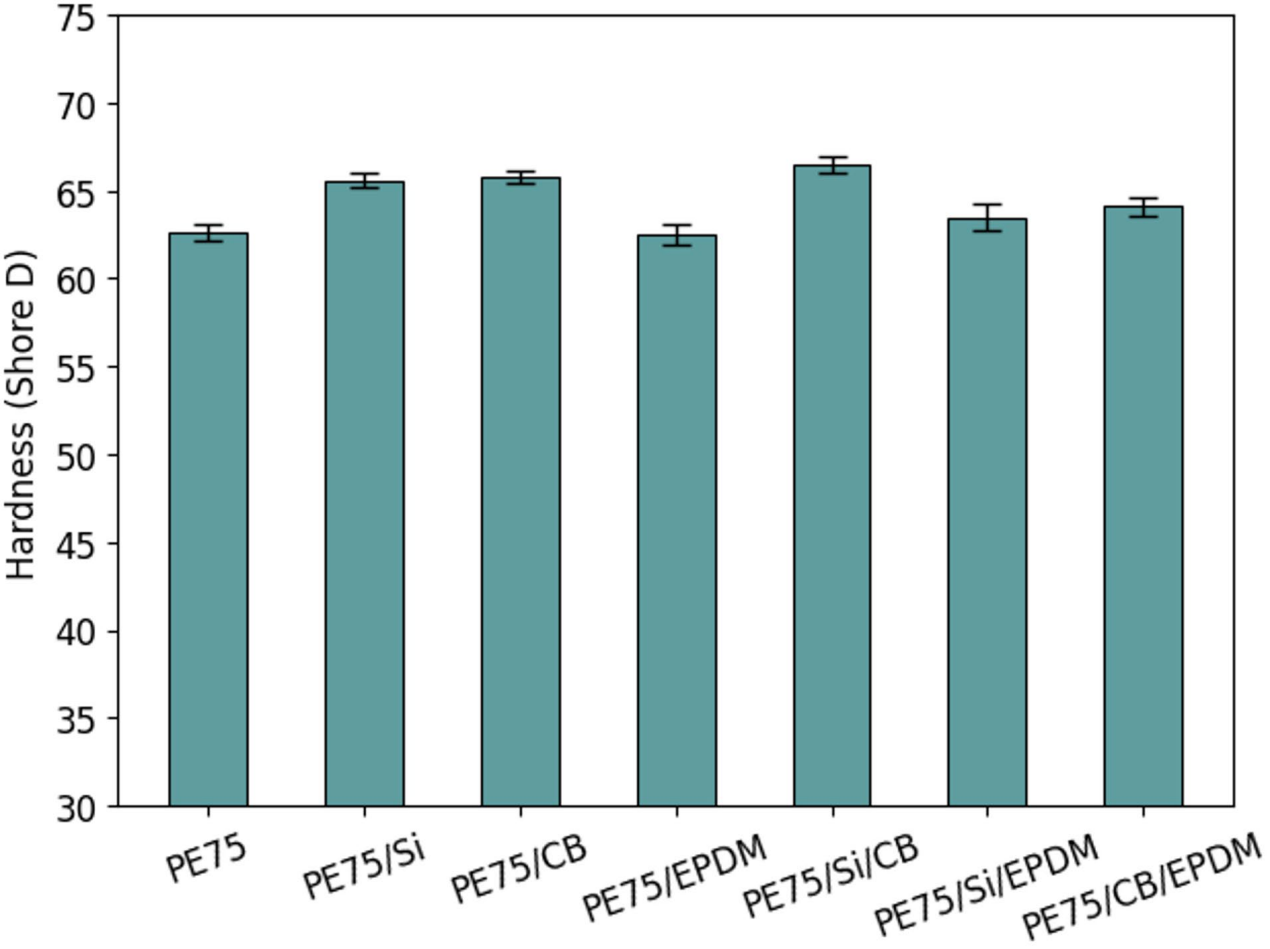
Hardness for neat PE75 blend and its micro-filled blends.

The effect of micro-scale additives on the mechanical performance of the PE75 blend is investigated. Silica, carbon black, EPDM, and their hybrid combinations were incorporated at a total loading of 5 wt% to evaluate and compare their influence on mechanical properties. The incorporation of these additives into the PE75 blend produced significant changes in its tensile behavior, influencing yield strength, stiffness, ductility, and toughness. Figure 5 displays the stress-strain curves for neat PE75 blend and its micro-filled blends. Figure 6 shows yield stress and modulus of elasticity for micro-filled blends. Figures 7 and 8 display elongation at break, toughness and hardness for micro-filled blends, respectively. Among all individual additives, silica provided a significant overall improvement. The yield strength increases by 17%, while the modulus of elasticity rises slightly by 2%. More pronounced improvements are observed in ductility and energy absorption, the elongation increases by 43% and toughness increases by 72%. In addition, hardness increases to 65.6 (5% increase), indicating effective rigid-particle reinforcement and improved resistance to surface deformation. This improvement can be attributed to good particle dispersion and efficient stress transfer within the matrix. Table 3 Summarizes the mechanical properties of PE75, and its micro-filled blends as mean values accompanied by standard deviations.

**Table 3:** Mechanical properties of PE75 blend and its micro-filled blends.

Property / composition	Yield strength (Mpa)	Modulus of elasticity (Mpa)	Elongation at break (%)	Toughness (MJ/m^3^)	Hardness (Shore D)
PE75	22.7 ± 0.3	203.5 ± 4.3	81.9 ± 9.4	12.1 ± 2.4	62.6 ± 0.5
PE75/Si	26.6 ± 0.4	208 ± 10.3	117.1 ± 10	20.8 ± 1.7	65.6 ± 0.4
PE75/CB	27.1 ± 0.6	221.7 ± 3.9	87.8 ± 9.8	16.3 ± 2.4	65.8 ± 0.4
PE75/EPDM	20.8 ± 0.2	199.3 ± 14.7	47.9 ± 6.2	7.6 ± 0.8	62.5 ± 0.6
PE75/Si/CB	28.4 ± 0.9	214 ± 13	36.8 ± 4.2	7.7 ± 0.6	66.5 ± 0.4
PE75/Si/EPDM	23.9 ± 0.9	224 ± 8.6	61 ± 9.4	10.2 ± 2.5	63.5 ± 0.7
PE75/CB/EPDM	23.2 ± 0.7	219.8 ± 17.8	36.3 ± 4.7	6.6 ± 1.2	64.1 ± 0.5

For the carbon black–filled blend, the yield strength increases by 19%, and the modulus of elasticity rises by 9%, confirming its strong stiffening effect. However, elongation increases only slightly by 7%, and toughness increases by 35%, also the hardness increases by 5%. This shows that carbon black produces a stronger and stiffer material.

EPDM composition exhibited the opposite behavior. The yield strength drops to 20.8 MPa (8% reduction), and the modulus decreased to 199.3 MPa (2% reduction). Elongation and toughness are reduced to 47.9% and 7.6 MJ/m^3^, corresponding to decreases of 42% and 37%, respectively. The hardness remains nearly unchanged (62.5). The stress–strain response shows early failure after yielding, confirming that EPDM at 5% does not provide any reinforcement and instead reduces the tensile performance of the blend. The reduction in mechanical performance observed with EPDM addition can be attributed to the relatively low loading level used in this study. In addition, the absence of reactive grafting functionality may have limited its ability to enhance interfacial adhesion between the HDPE and PP phases. At such concentrations, EPDM may act as a dispersed soft phase without providing effective toughening or compatibilization. In contrast, previous studies have reported improvements at higher EPDM contents (up to 30–50 wt%), where a more pronounced rubber toughening effect is achieved^24^.

When mixing additives at 2.5 wt% and 2.5 wt%, the tensile response depended strongly on the pair used. The silica/carbon black hybrid exhibits the highest yield strength by 25% increase, and a moderate increase in modulus of elasticity by 5%. However, elongation decreases sharply by 55%, and toughness reduces by 36%, indicating a transition toward brittle behavior. This composition also shows the highest hardness 66.5 (6% increase), consistent with its stiff nature.

The silica/EPDM hybrid displays a more balanced response, with yield strength increasing to by 5% increase and modulus of elasticity rising by 10%. Elongation at break decreases by 26%, while toughness reduces by 16%. reflecting partial compensation between silica reinforcement and EPDM softening.

For the carbon black/EPDM hybrid, the yield strength increases slightly by 2% and the modulus of elasticity increases by 8%. However, elongation and toughness decrease significantly by 56% and 46%, respectively. The hardness slightly increases to 64.1 (2% increase), indicating increased stiffness accompanied by reduced ductility.

Overall, maintaining a constant additive content of 5% clearly shows that silica provides the best overall enhancement to the PE75 blend, where a significant increase in yield strength, ductility and toughness is observed among all tested compositions. These results indicate that silica interacts more favorably with the blend structure, and its balanced performance strongly motivates further investigation into nano-silica as a promising route for additional mechanical enhancement of the PE75 blend. It should be noted that the additives used in this study are not intended to act as reactive compatibilizers, but rather to enhance the properties through physical interactions and improved stress transfer within the blend.

#### Polymer blend with silica nano particles

**Fig. 9:**
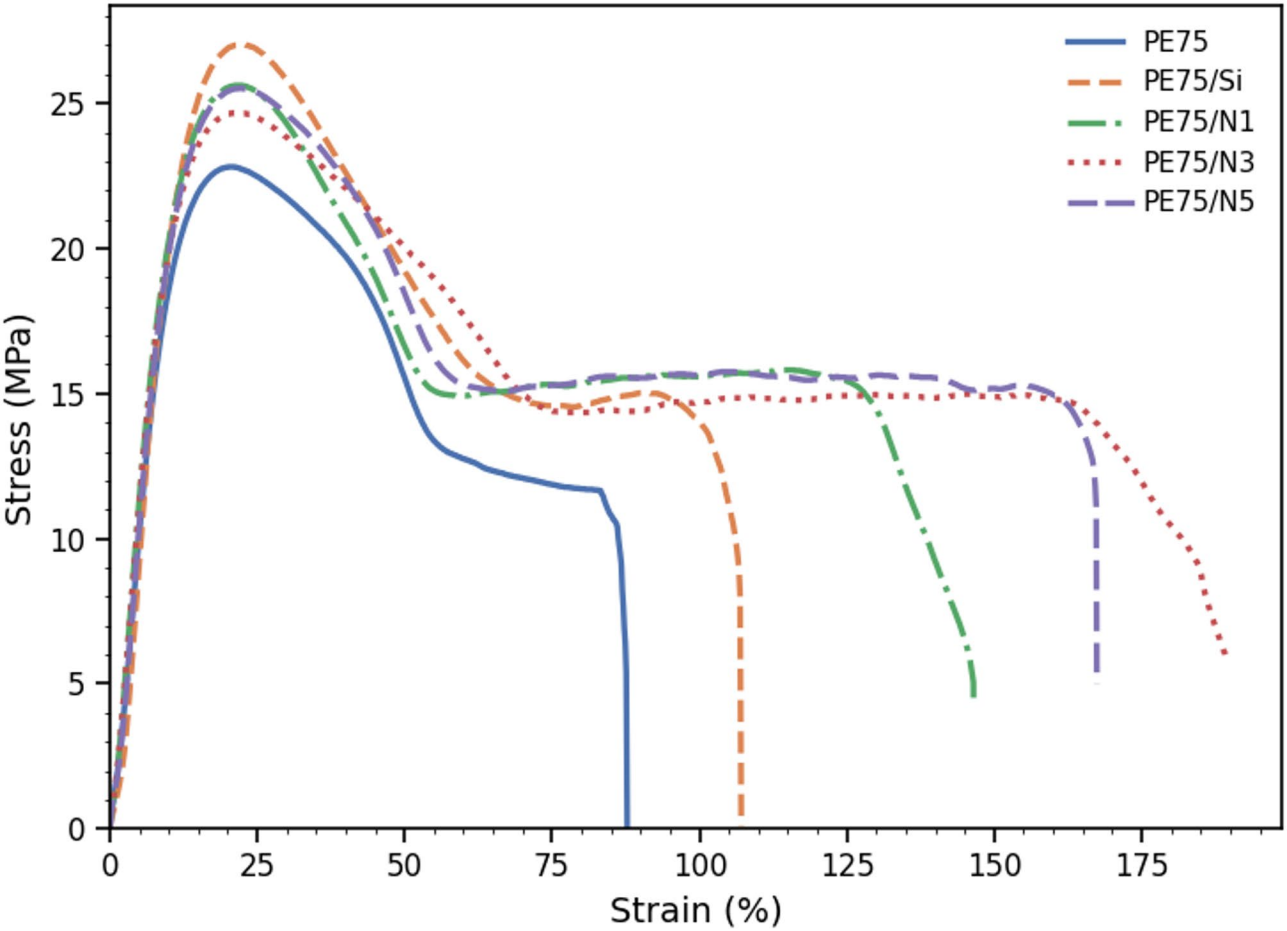
Stress-strain curves for neat PE75, and PE75 with silica and nano-silica at different ratios.

**Fig. 10:**
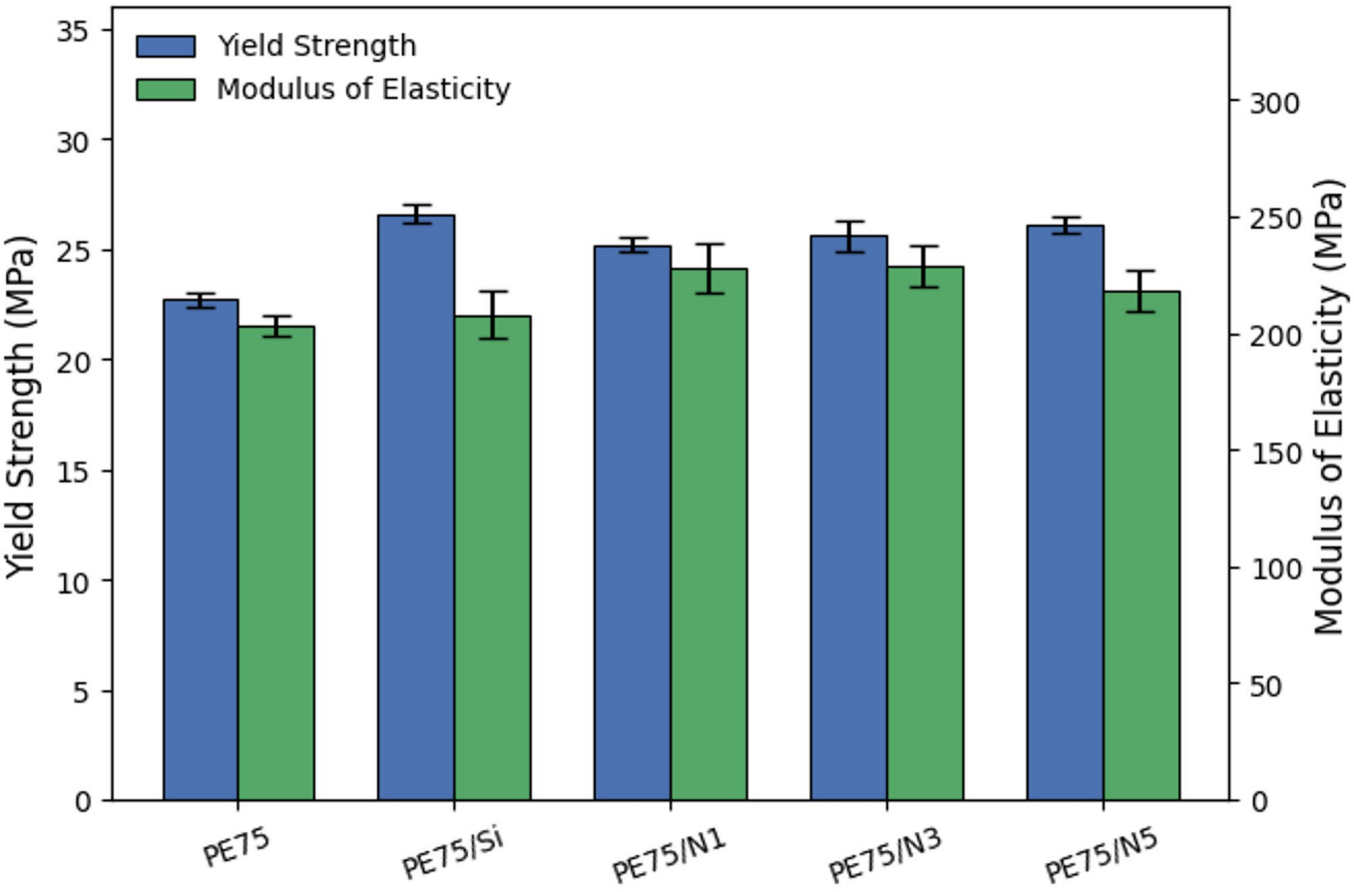
Yield stress and modulus of elasticity for neat PE75, and PE75 with silica and nano-silica at different ratios.

**Fig. 11:**
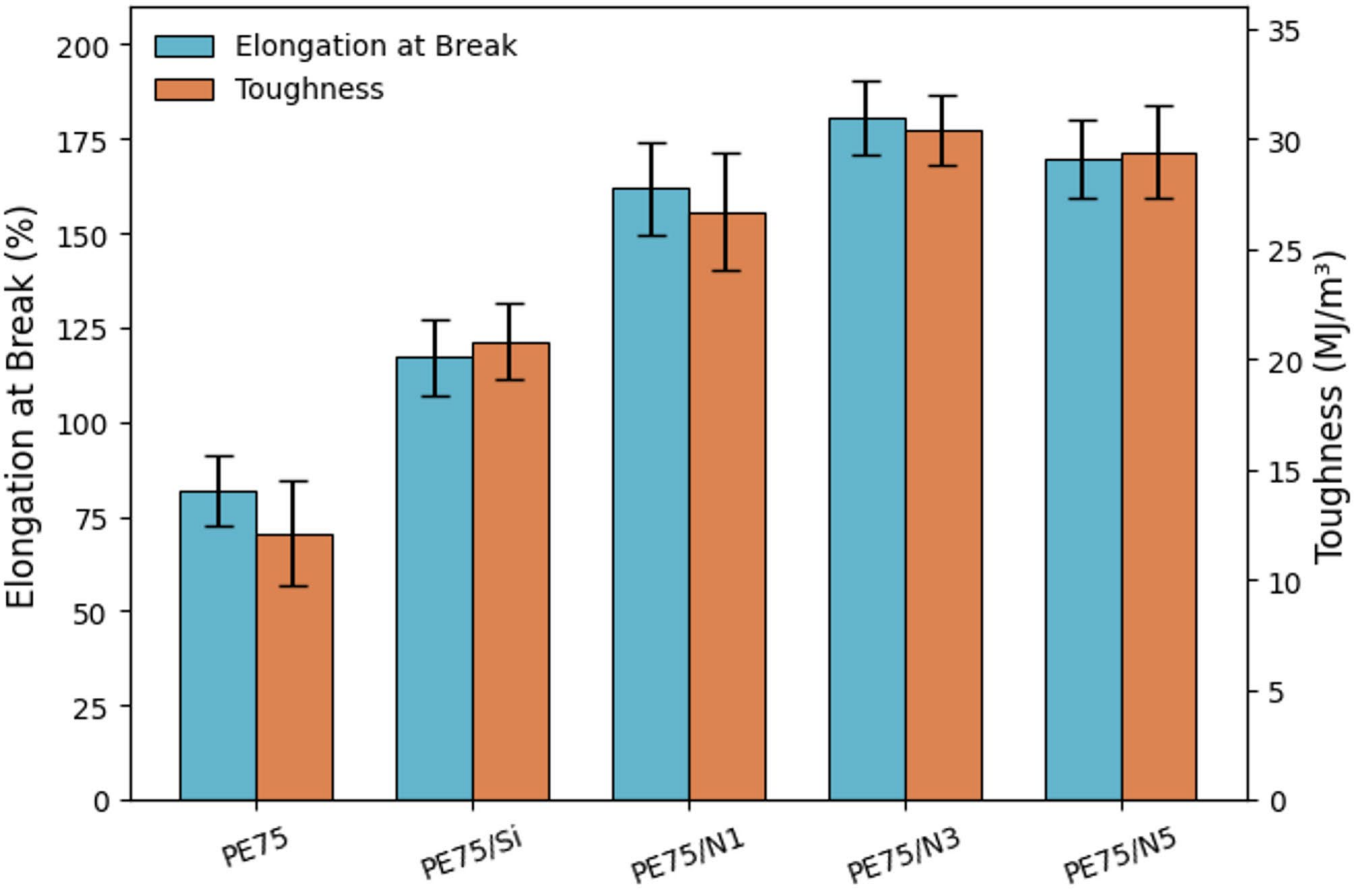
Elongation at break and toughness for neat PE75, and PE75 with silica and nano-silica at different ratios.

**Fig. 12:**
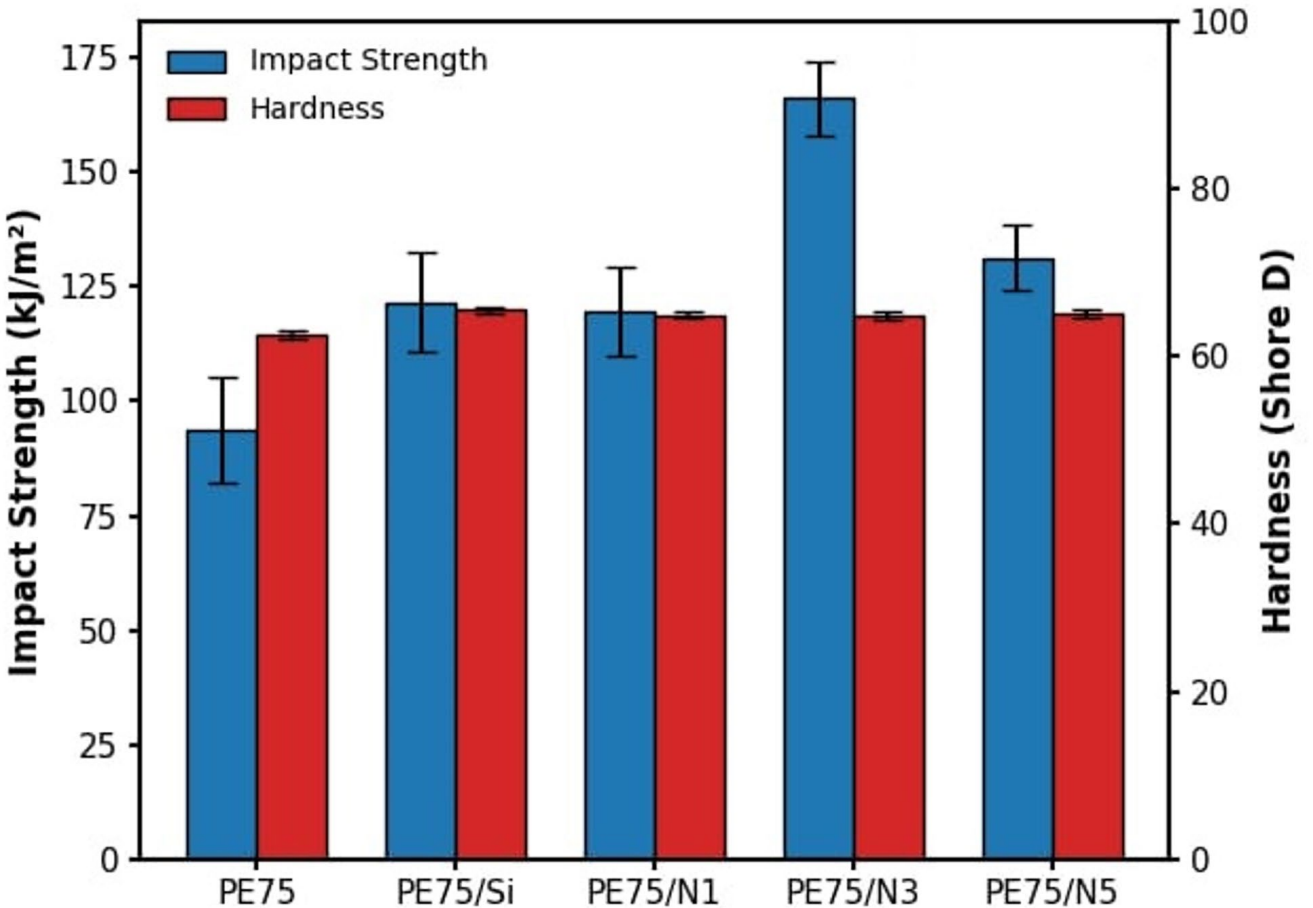
Impact strength and hardness for neat PE75, and PE75 with silica and nano-silica at different ratios.

Nano-silica were investigated over a loading ratio of 1, 3 and 5 wt% to identify the optimum content. Due to their high surface area, nano-particles can significantly enhance interfacial interactions at low concentrations; however, higher loadings may promote agglomeration, adversely affecting dispersion and overall performance. These concentrations were selected to identify the optimal percentage that maximizes mechanical enhancement while minimizing filler content. Figure 9 shows the stress-strain curves for neat PE75, and PE75 with silica and nano-silica at different ratios. Figure 10 shows yield stress and modulus of elasticity for the compositions. Compared with the neat blend, all nano-silica formulations showed higher yield strength, increased stiffness, and remarkable gains in elongation and toughness. This reflects the strong reinforcing capability of nano-sized particles due to their high surface area and improved interfacial interaction with the polymer matrix^38^. Figure 11 represent the elongation at break and toughness for neat PE75, and PE75 with silica and nano-silica at different ratios. Figure 12 represent the impact strength and hardness for neat PE75, and PE75 with silica and nano-silica at different ratios. Table 4 presents the mechanical properties of PE75 with silica and nano-silica at different ratios as mean values accompanied by standard deviations.

**Table 4:** Mechanical properties of PE75 with silica and nano-silica at different ratios.

Property / Composition	Yield Strength (Mpa)	Modulus of Elasticity (Mpa)	Elongation at Break (%)	Toughness (MJ/m3)	Hardness (Shore D)	Impact strength (kJ/m^2^)
PE75	22.7 ± 0.3	203.5 ± 4.3	81.9 ± 9.4	12.1 ± 2.4	62.6 ± 0.5	93.9 ± 11.6
PE75/Si	26.6 ± 0.4	208 ± 10.4	117.1 ± 10	20.8 ± 1.7	65.6 ± 0.4	121.6 ± 10.9
PE75/N1	25.2 ± 0.3	227.8 ± 10.8	161.9 ± 12.2	26.7 ± 2.7	65 ± 0.4	119.7 ± 9.6
PE75/N3	25.6 ± 0.7	229.1 ± 8.7	180.6 ± 9.8	30.4 ± 1.6	64.9 ± 0.5	166.2 ± 8.1
PE75/N5	26.1 ± 0.4	218.5 ± 8.8	169.8 ± 10.5	29.4 ± 2.1	65.2 ± 0.5	131.4 ± 7.3

At 1% nano-silica, the blend shows a rise in modulus of elasticity by 12%, along with an improvement in yield strength by 11%. More notably, elongation at break increased by 98% and more pronounced enhancement in toughness by 120% increase.

At 3% nano-silica, further improvements are observed. The modulus of elasticity and yield strength both increase by 13%, elongation and toughness increase significantly by 120% and 150%, respectively. The stress–strain curve reflects a well-developed strain-hardening region and delayed failure, suggesting excellent load transfer and uniform particle dispersion at this concentration.

At 5% nano-silica, the yield strength and modulus of elasticity increased by 15% and 8%, respectively. The elongation at break and toughness increased by 107% and 143%, respectively, compared to the neat blend. The slight drop compared with the composition of 3% nano silica suggests the beginning of particle agglomeration^39^, which may limit the efficiency of reinforcement at higher loadings.

In conclusion, nano-silica offers greater performance enhancement than micro-silica, particularly in ductility and toughness, while all nano-silica blends show similar hardness levels (~ 65 Shore D), comparable to those achieved with micro-silica. The improvements achieved clearly demonstrate the efficiency of nano-scale reinforcement, where small concentrations produce substantial gains in mechanical performance. The observed improvement at low nano-silica loading is attributed to effective dispersion and enhanced interfacial interaction, as reported by^39^. These results confirm that nano-silica is an effective modifier for the PE75 blend, offering remarkable increases in strength, ductility, and toughness. The low nano-silica loading (≤ 3 wt%) and its commercial availability at relatively low cost support the feasibility of this approach for large-scale recycling, although proper dispersion remains essential to ensure consistent performance.

The impact strength of the PE75-based blends exhibits a significant dependence on nano-silica incorporation. The neat PE75 blend shows an impact strength of 93.9 kJ/m², which increases by approximately 29.5% with the addition of silica (121.6 kJ/m²), indicating an improvement in energy absorption capability. Upon introducing nano-silica at low loading (1 wt%), the impact strength (119.7 kJ/m²) remains comparable to PE75/Si. A pronounced enhancement is observed at loading 3 wt%, where the impact strength reaches 166.2 kJ/m², representing an increase of approximately 77% compared to the neat blend. This significant improvement is attributed to effective stress transfer and enhanced energy dissipation mechanisms due to well-dispersed nanoparticles. However, further increasing the nano-silica content to 5 wt%, results in a reduction in impact strength to 131.4 kJ/m², corresponding to a decrease of about 21% compared to 3 wt% composition, although it still remains higher than the neat blend by approximately 39.9%. This reduction at higher loading is likely due to nanoparticle agglomeration and the formation of stress concentration sites that facilitate premature fracture. Overall, the results indicate the presence of an optimal nano-silica loading (3 wt%) maximizes toughness, after which no further improvement observed.

#### Finite element modeling

**Fig. 13:**
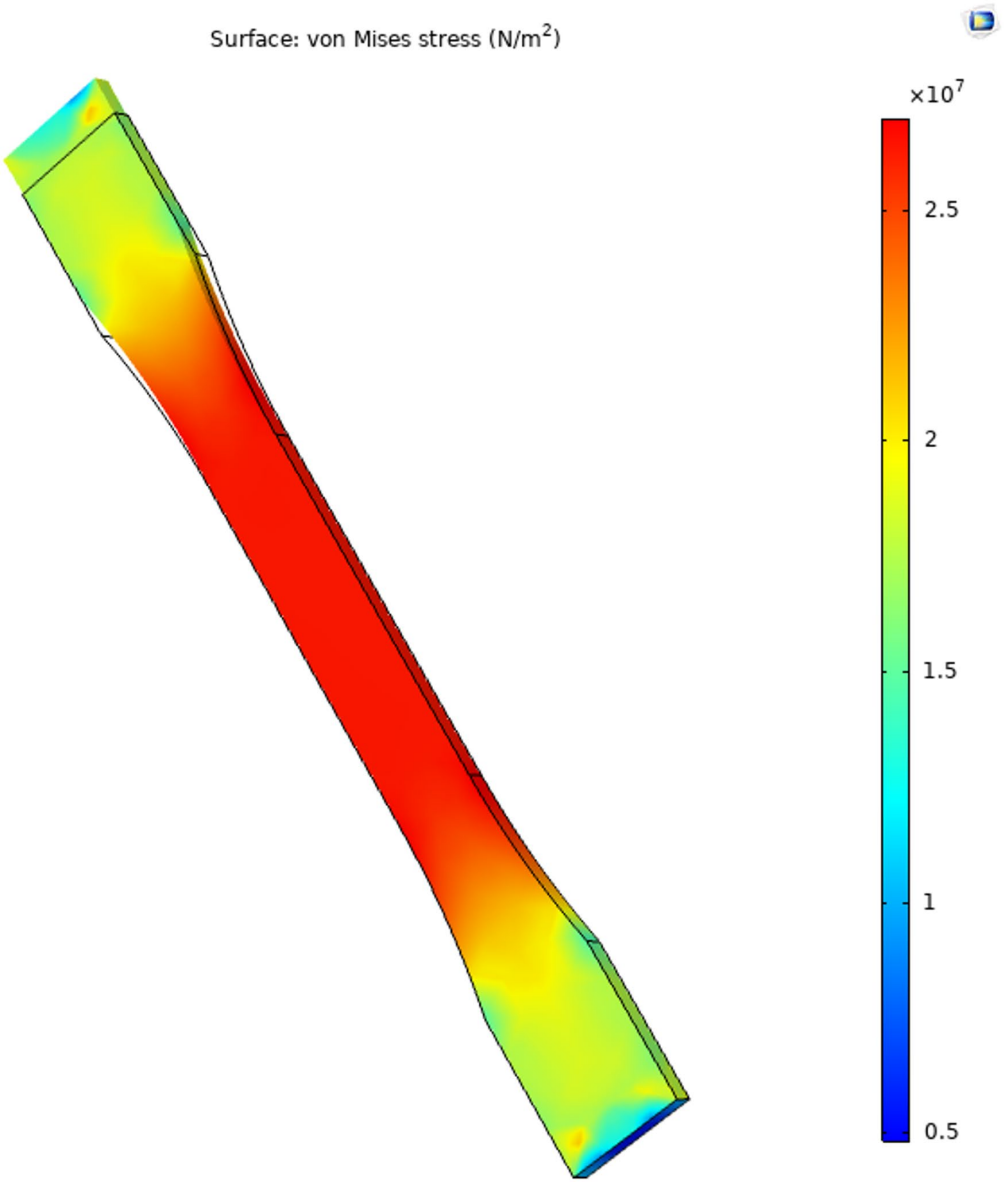
Von Mises stress distribution in the ASTM D638 Type-I tensile specimen under uniaxial loading.

**Fig. 14:**
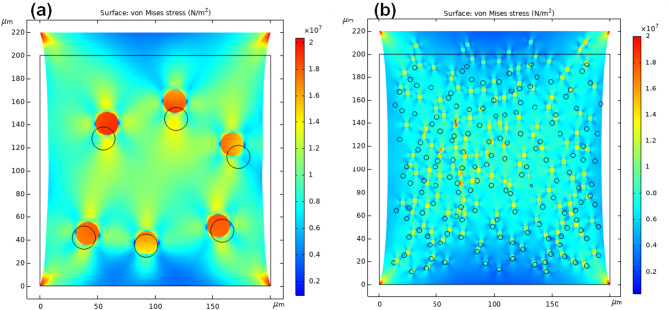
Von Mises stress distribution in PE75 composites reinforced with (**a**) micro-silica and (**b**) nano-silica.

COMSOL Multiphysics was employed to simulate the influence of micro and nano-scale silica, each incorporated at 5 wt%, on the mechanical response of the PE75 composite under uniaxial tension. Figure 13 illustrates the von Mises stress distribution in the tensile specimen under uniaxial loading, showing maximum stresses concentrated in the gauge region, where fracture is experimentally observed. Figure 14 presents the simulated stress fields for the micro-silica and nano-silica reinforced composites. To quantify the effect of filler size on stress distribution, the stress concentration factor (SCF) was evaluated from the COMSOL simulations as the ratio between the maximum local stress and the nominal applied stress. The micro-silica-reinforced composite exhibits a relatively high SCF of approximately 3.6, indicating pronounced stress localization around the larger particles and polymer–filler interfaces, which can limit mechanical improvement. In contrast, the nano-silica composite shows a significantly lower SCF of about 2.4, reflecting a more uniform stress distribution and reduced stress concentration, reflecting efficient stress transfer and stronger interfacial interaction. This homogeneous stress field delays damage initiation and supports stable plastic deformation, which is consistent with the significantly enhanced elongation and toughness observed experimentally. The finite element results confirm that improved filler dispersion leads to more uniform stress distribution and superior mechanical performance, explaining the outstanding reinforcing efficiency of nano-silica in the PE75 blend. The simulation was performed under several simplifying assumptions. The materials were considered homogeneous and isotropic. The particle geometry and distribution were idealized, and the analysis was conducted under linear elastic conditions. A limited displacement was applied to represent the initial elastic region of the material before yielding. However, these assumptions reasonably reflect the general morphological features observed experimentally and provide qualitative insight into the stress transfer behavior. The material properties used in the simulation were based on experimentally measured values for the polymer matrix, while literature data were adopted for filler properties where direct measurements were not available. It should be noted that the calculated SCF values are sensitive to particle size distribution and interfacial properties; however, the model captures the general trends in stress distribution between micro- and nano-filled systems.

### Morphological analysis

**Fig. 15:**
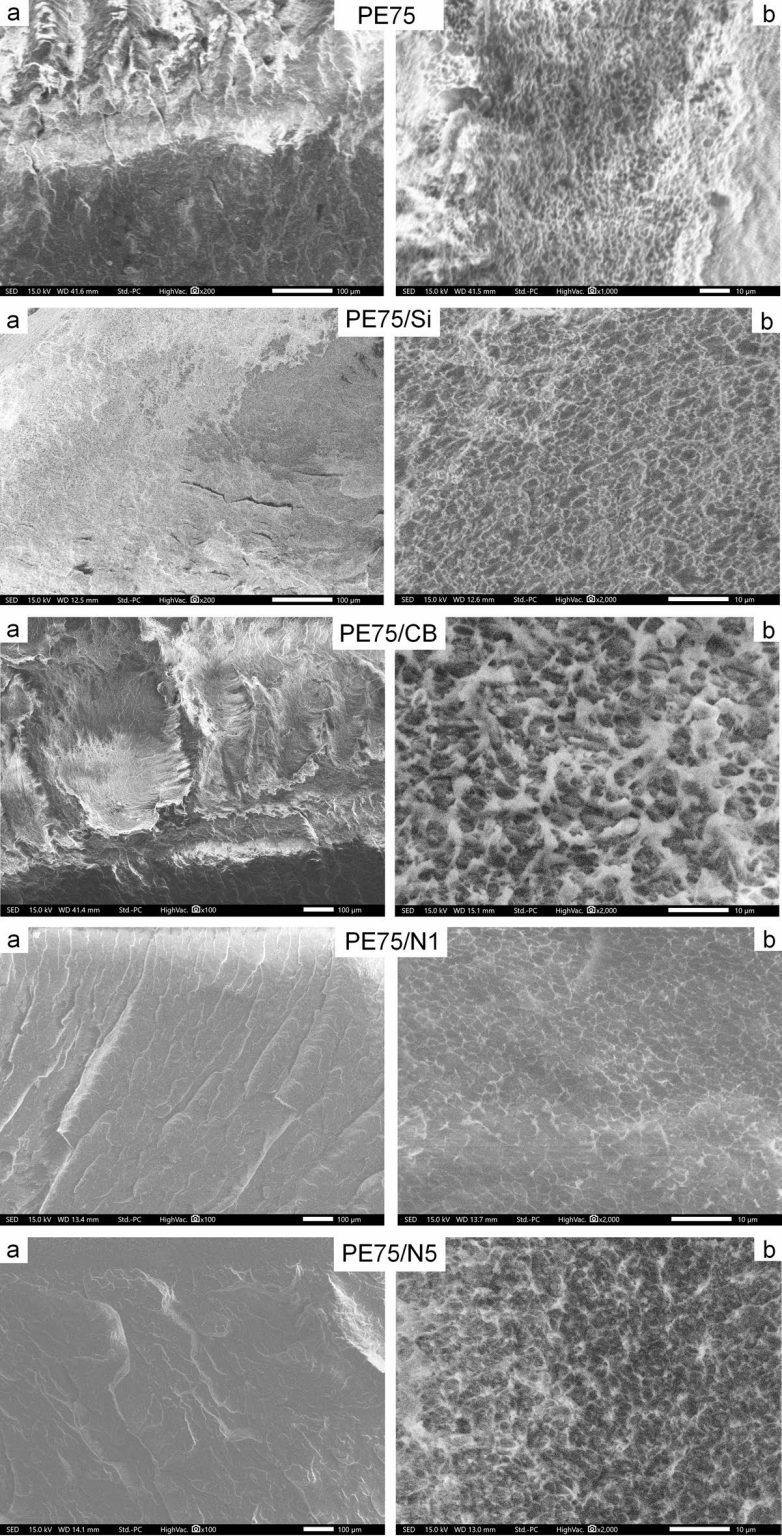
SEM micrographs for different compositions with scale bars: (**a**) 100 μm, (**b**) 10 μm.

SEM micrographs of the cryo-fractured surfaces of the neat blend and its filler-modified compositions are presented at different magnifications in Fig. 15. Due to limitations in SEM availability, only the representative and most informative compositions were selected for detailed microscopic comparison. The fractured surface of the PE75 neat blend exhibits the typical morphology of immiscible PE/PP systems, where the PE-rich continuous matrix undergoes extensive shear deformation during cryofracture. Smooth tearing lines, shear bands, and well-developed fibrillation can be observed, reflecting the ductility of the material. The PP phase appears as dispersed domains embedded within the PE matrix, consistent with the limited compatibility between the two polymers.

Upon incorporation of 5 wt% silica, the morphology becomes noticeably more refined and uniform. SEM images show that the silica particles are well embedded within the matrix, with minimal signs of particle pull-out or interfacial voids. The presence of micro-silica appears to stabilize the PE/PP interface and reduce the size of PP domains, promoting a more homogeneous two-phase morphology. The absence of large voids or brittle fracture features indicates good polymer–filler adhesion and efficient stress transfer, which supports the observed enhancement in mechanical properties.

Carbon black also produces a distinctive morphology but with less uniform distribution than micro-silica. SEM micrographs reveal fine CB aggregates dispersed throughout the matrix, with micro voids and small pull-out pits. Although these aggregates can act as stress concentrators, their relatively small size and good embedding indicate an acceptable interfacial interaction. This microstructure corresponds to the mechanical behavior, where carbon black increases yield strength.

A significant improvement in morphological refinement is observed with the addition of nano-silica. At 1 wt% nano-silica, the surface becomes noticeably more refined. At higher magnification images show that the PE/PP domains are significantly smaller and more uniformly distributed, indicating that the nano-silica was well dispersed throughout the matrix. No voids, pull-out pits, or agglomerates are visible, suggesting strong polymer–particle interaction. This refined morphology enhances stress transfer without restricting molecular mobility, which explains the significant improvement in ductility and toughness.

At 5 wt% nano-silica, minor signs of clustering appear, visible as small silica-rich zones. However, these clusters remain relatively limited in size, and the fracture surface still shows cohesive tearing with minimal void formation. This explains why the mechanical properties remain significantly better than the neat and micro-filled blends, despite a slight reduction relative to the 3% sample.

Quantitative analysis of the phase morphology was performed using ImageJ software, where the average domain size was calculated based on measurements of at least 40 domains for each composition, and the resulting domain size statistics are reported in Table 5. The analysis reveals a clear dependence of phase morphology on both the type and loading of additives. The neat blend exhibits the largest domain size, with carbon black showing only a marginal effect. Micro-silica provides moderate refinement, while nano-silica leads to a significant reduction in domain size, reaching a minimum size at 1 wt% loading and slightly increasing at 5 wt% due to partial agglomeration.

**Table 5:** Average domain size of compositions.

Composition	Domain size (µm)
PE75	3.2 ± 0.8
PE75/Si	2.4 ± 0.6
PE75/CB	3.0 ± 0.7
PE75/N1	1.4 ± 0.4
PE75/N5	1.9 ± 0.5

The results demonstrate how distinct modification mechanisms influence the interface between PE and PP phases. In particular, nano-silica enhance interfacial interactions by promoting finer phase dispersion and reducing domain size, indicating improved interfacial adhesion. In contrast, micro-scale fillers show a more limited effect. Furthermore, the incorporation of morphology analysis allows for direct correlation between domain size and mechanical performance, providing insight into the relationship between interfacial structure and macroscopic properties.

Overall, the SEM observations clearly reflect the mechanical behavior. Well-dispersed nano silica significantly refines the PE/PP morphology, strengthen interfacial adhesion that support enhanced mechanical properties. The resulting morphology facilitates more efficient stress transfer and delayed crack propagation, leading to enhanced toughness. In particular, low-loading nano-silica produces an exceptionally uniform and ductile microstructure, confirming its superior reinforcing efficiency.

### Thermogravimetric analysis (TGA)

#### Thermal degradation behavior of neat PE/PP blends

**Fig. 16:**
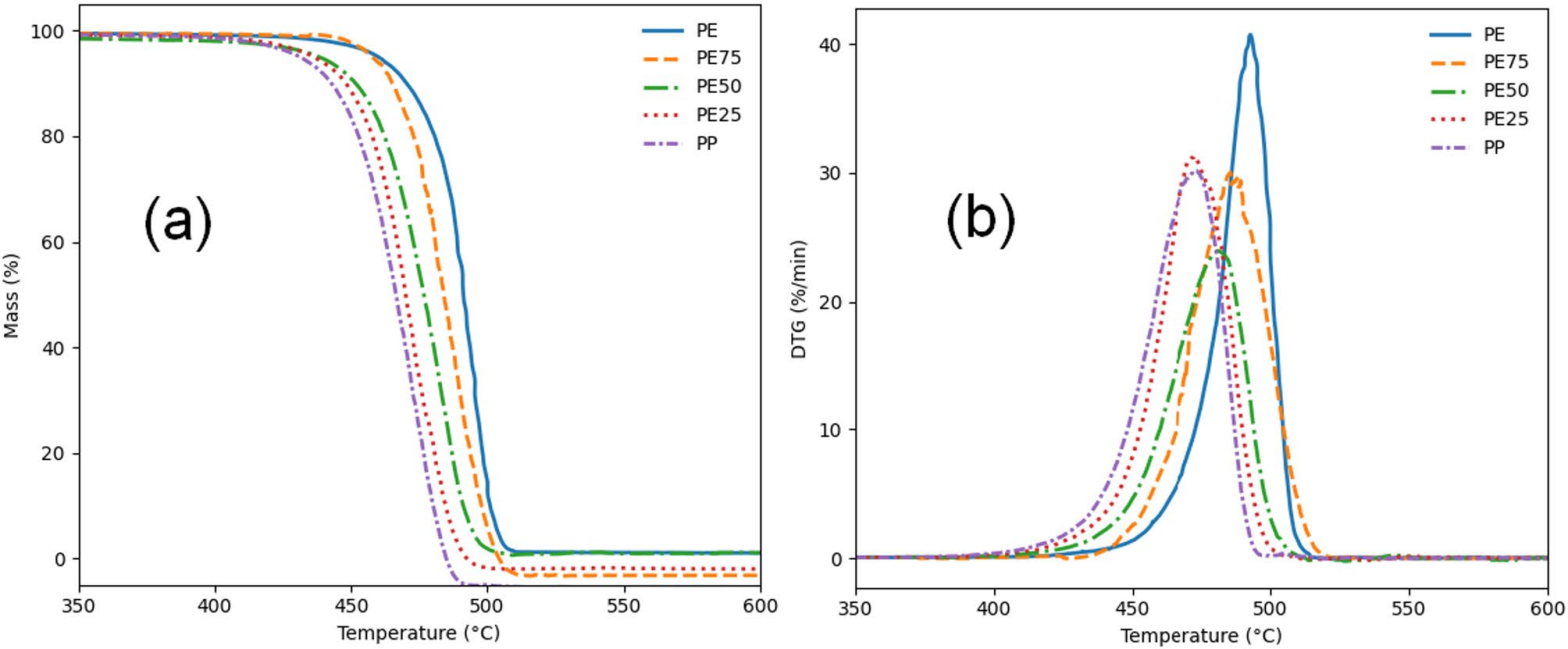
(**a**) TG and (**b**) DTG curves for of PE, PP, and neat blends.

**Fig. 17:**
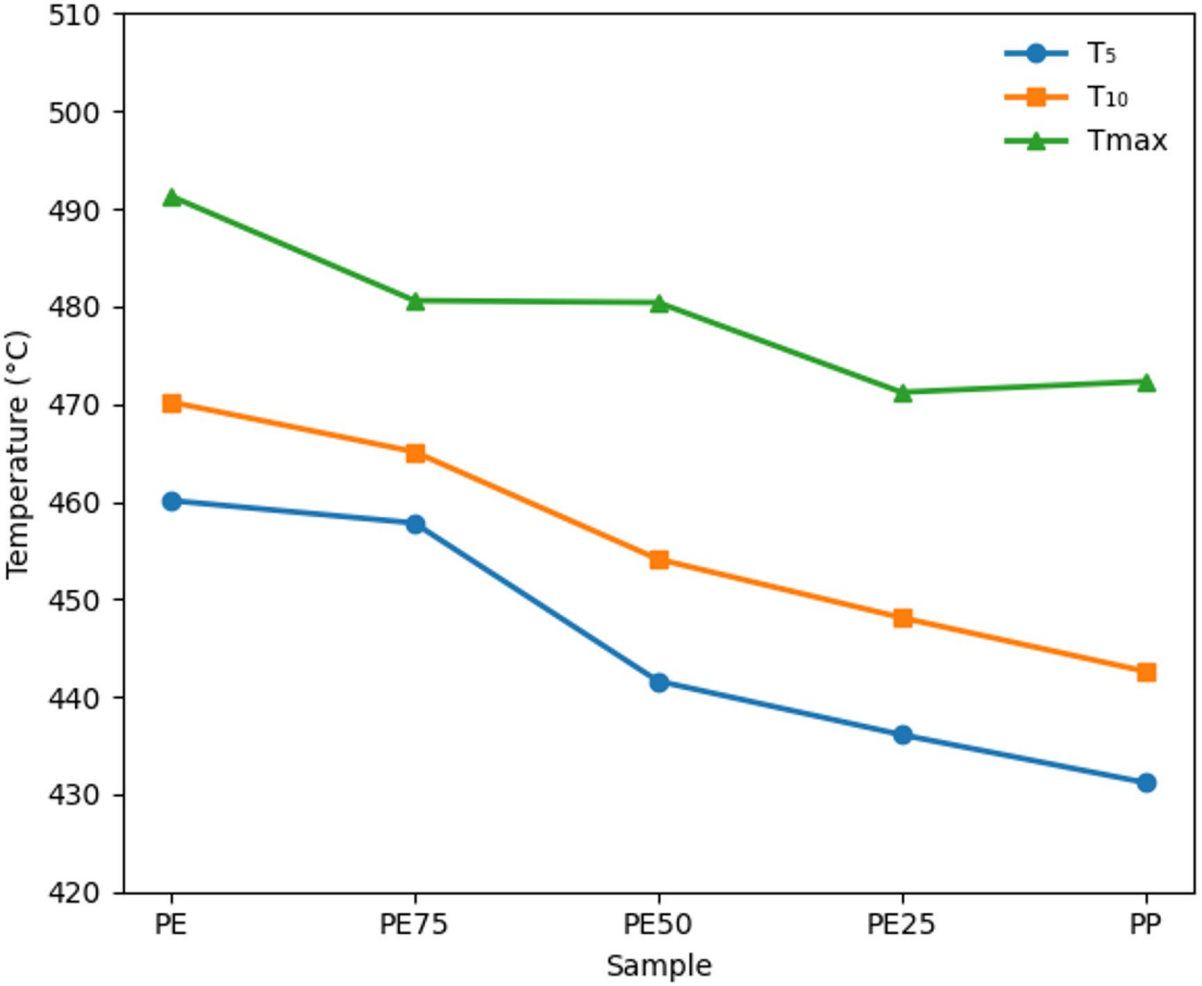
Degradation temperatures (T₅, T₁₀, and T_max_) for PE, PP, and neat blends.

The thermal degradation behavior of neat polyethylene (PE), polypropylene (PP), and their binary blends was investigated by thermogravimetric analysis under inert atmosphere. The TG and DTG curves of PE, PP, and PE/PP blends with varying PE content are presented in Fig. 16, while the characteristic degradation temperatures are illustrated in Fig. 17.

Neat PE exhibited the highest thermal stability, with T₅ = 460.1 °C, T₁₀ = 470.2 °C, and T_max_ = 491.3 °C, due to its linear structure and absence of tertiary carbon atoms. In contrast, PP showed the lowest stability (T₅ = 431.2 °C, T₁₀ = 442.6 °C, T_max_ = 472.3 °C) as degradation is governed by chain scission at tertiary carbons^13^. The T_10_ values were evaluated to further verify the onset degradation trend. All compositions showed a comparable shift in T_10_ towards higher temperatures, consistent with T_5_ values.

PE75 showed a slight reduction (T₅ = 457.8 °C, T_max_ = 480.6 °C), while PE50 exhibited a more pronounced drop in T₅ (441.6 °C) with T_max_ remaining ~ 480.4 °C, indicating earlier degradation onset but overlapping degradation behavior. Further decrease was observed for PE25 (T₅ = 436.1 °C, T_max_ = 471.2 °C), where PP dominates the thermal response.

Overall, increasing PP content reduces thermal stability, mainly affecting the degradation onset. All samples showed a single-step degradation with no residual char, typical of polyolefins under inert atmosphere.

#### Thermal degradation behavior of PE75 blend with micro particles

**Fig. 18:**
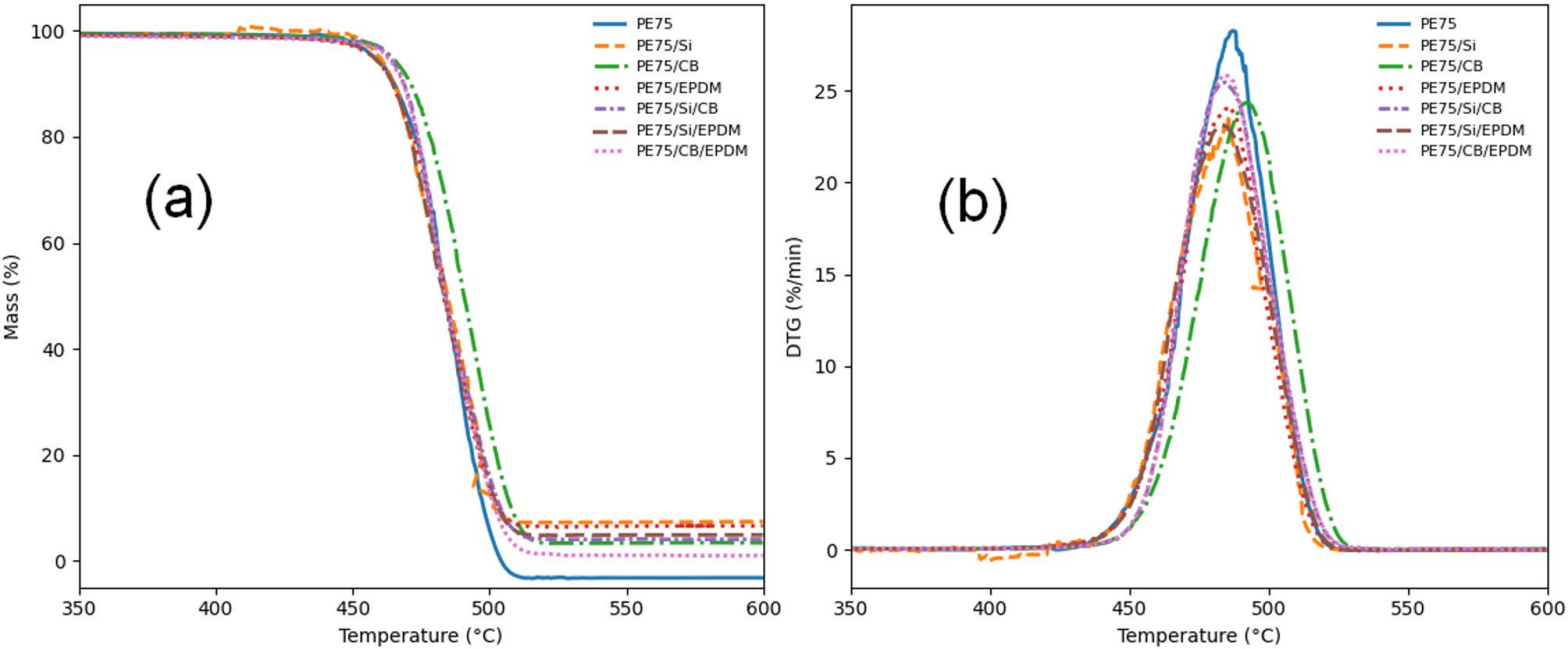
(**a**) TG and (**b**) DTG curves for of PE75 and micro-filled blends.

**Fig. 19:**
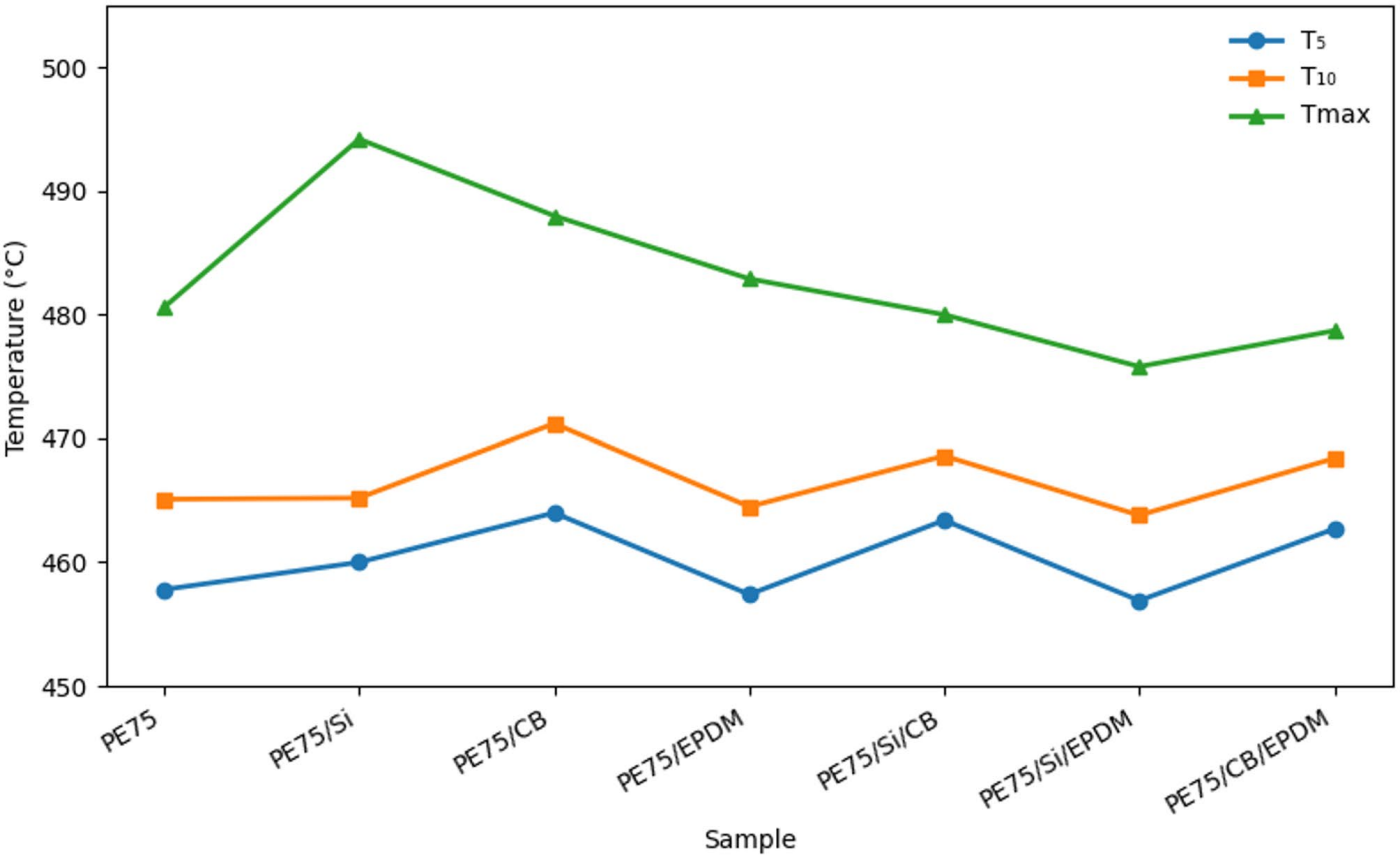
Degradation temperatures (T₅, T₁₀, and T_max_) for PE75 and micro-additive composites.

**Fig. 20:**
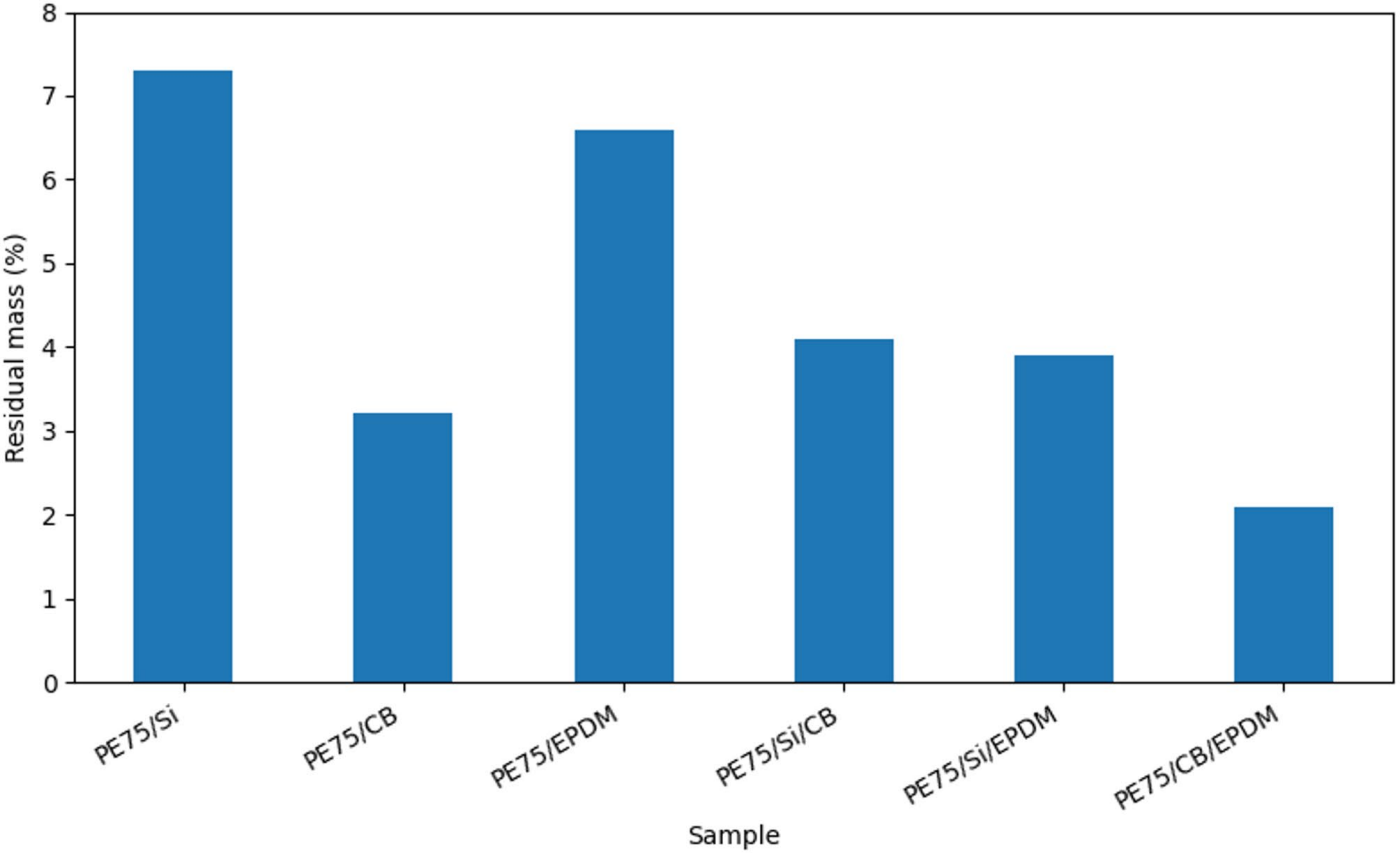
Residual mass percentage for PE75-based systems containing micro-additives.

The influence of micro-scale additives on the thermal degradation behavior of the PE75 blend was evaluated and the corresponding TG and DTG curves are presented in Fig. 18. The characteristic degradation temperatures and residual masses are presented in Figs. 19 and 20, respectively. For comparison, the thermal parameters of the unmodified PE75 blend are also included.

PE75/Si slightly improved thermal stability, increasing T₅ to 460.0 °C and significantly shifting T_max_ to 494.2 °C, attributed to its barrier effect. A high residual mass (7.3%) confirms the presence of inorganic filler. PE75/CB showed stronger improvement at early degradation stages (T₅ = 464.0 °C, T₁₀ = 471.2 °C), with a moderate increase in T_max_ (488.0 °C) and a residual of 3.2%, reflecting its thermal stability.PE75/EPDM had minimal effect on onset temperatures, with a slight increase in T_max_ (482.9 °C). The residual (6.6%) is linked to partial carbonization of the elastomer.

PE75/Si/CB increased T₅ (463.4 °C) but slightly reduced T_max_ (480.0 °C), indicating delayed onset without strong stabilization of main degradation. PE75/Si/EPDM showed reduced stability (T₅ = 456.9 °C, T_max_ = 475.8 °C), suggesting EPDM promotes earlier degradation despite silica presence. PE75/CB/EPDM exhibited higher T₅ (462.7 °C) but lower T_max_ (478.7 °C), indicating CB delays initiation while EPDM affects later stages; it also showed the lowest residue (2.1%). All systems maintained a single degradation peak, confirming unchanged degradation mechanism, with variations reflecting changes in degradation kinetics rather than new pathways.

#### Thermal degradation behavior of PE75 blend with nano silica particles

**Fig. 21:**
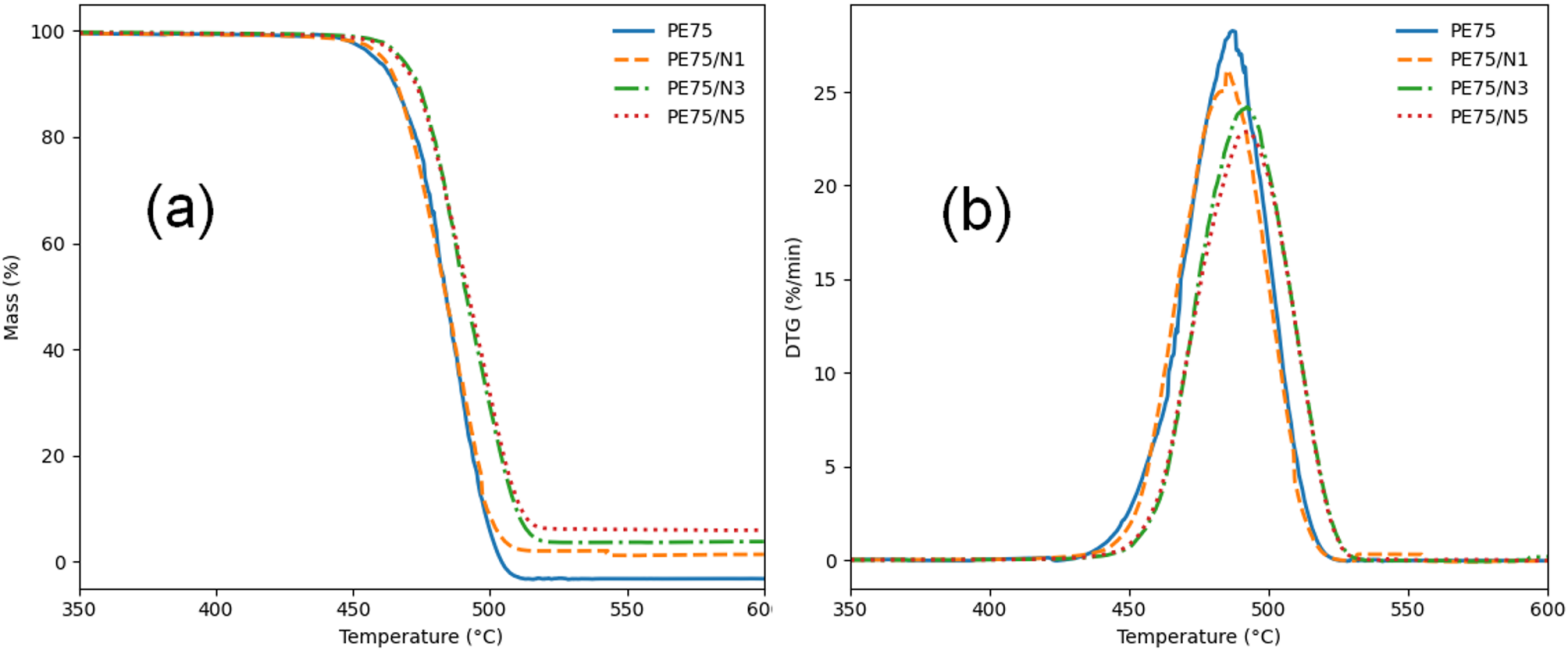
(**a**) TG and (**b**) DTG curves for of PE75 and nano-silica filled blends.

**Fig. 22:**
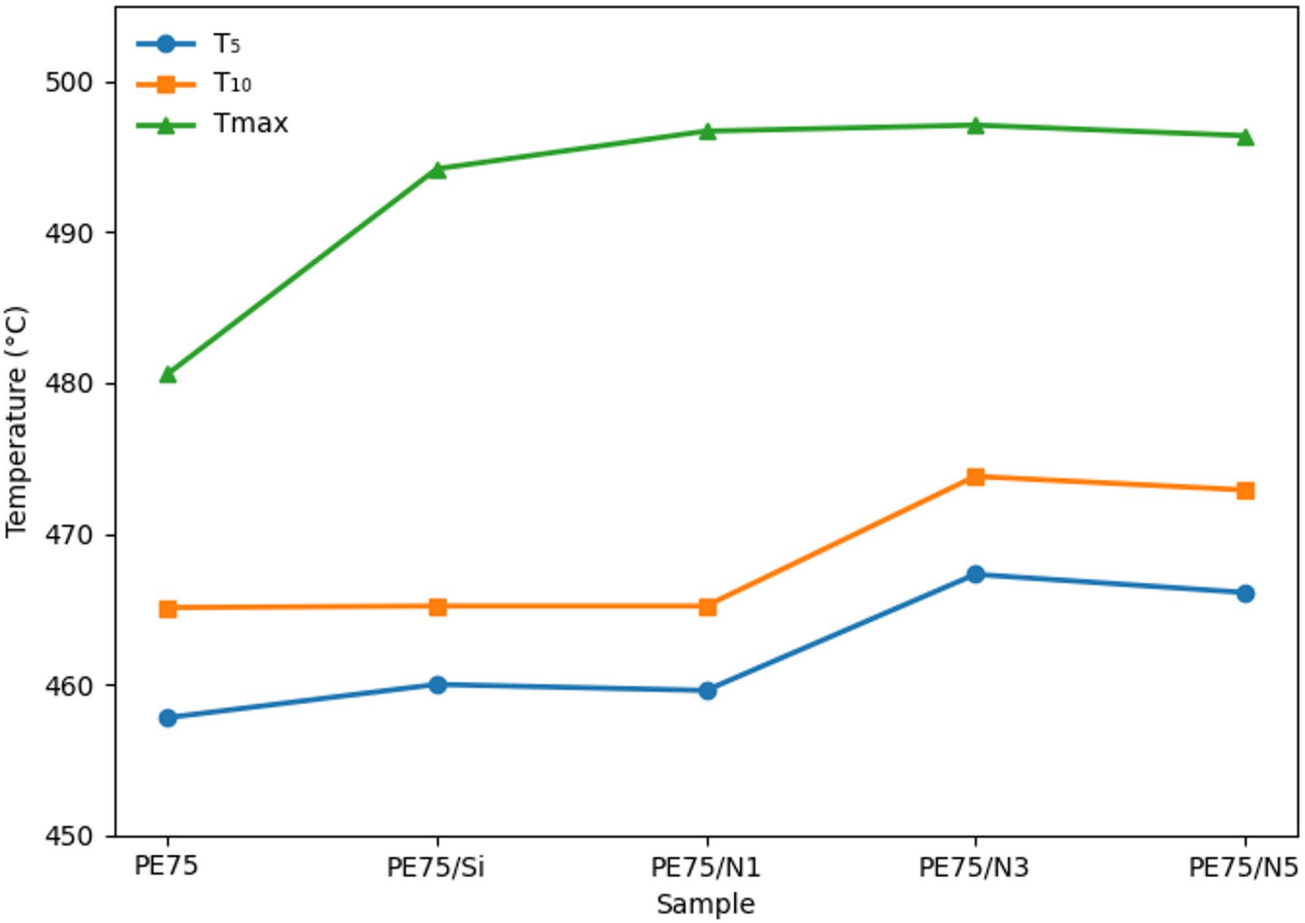
Degradation temperatures (T₅, T₁₀, and T_max_) for PE75, micro and nano-silica compositions at different ratios.

**Fig. 23:**
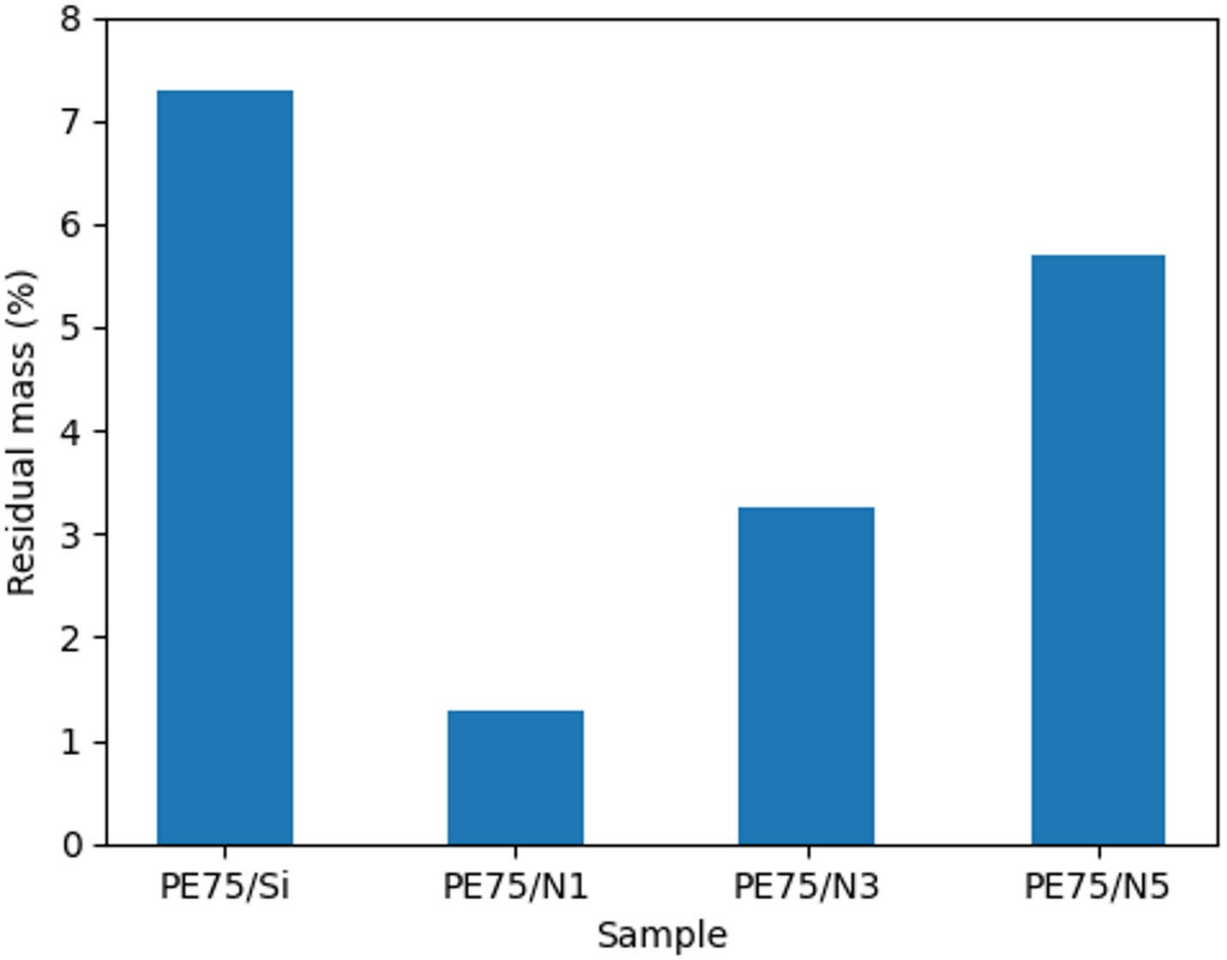
Residual mass for PE75-based systems containing micro and nano-silica at different ratios.

The effect of nano-silica incorporation on the thermal degradation behavior of the PE75 blend was investigated, and the corresponding TG and DTG curves are presented in Fig. 21. The characteristic degradation temperatures and residual masses are presented in Figs. 22 and 23, respectively.

Nano-silica incorporation improved the thermal stability of PE75 in a loading-dependent manner. At 1 wt%, a slight increase in T₅ (459.6 °C) and a significant rise in T_max_ (496.7 °C) indicate delayed main degradation. The effect becomes more pronounced at 3 wt%, where the highest values were obtained (T₅ = 467.3 °C, T_max_ = 497.1 °C), confirming effective retardation of both degradation onset and propagation. Increasing the loading to 5 wt% maintains high onset temperatures (T₅ = 466.1 °C) with a similar T_max_ (496.4 °C), suggesting a stabilization plateau at higher content. Compared to micro-silica, nano-silica provides greater enhancement, attributed to its higher surface area and better dispersion, which restrict chain mobility and diffusion of degradation products. All samples exhibited a single DTG peak shifted to higher temperatures, indicating unchanged degradation mechanism but slower kinetics. Overall, nano-silica significantly improves the thermal stability of the blend, as reflected by the increased degradation onset temperature and the shift of the main degradation peak toward higher temperatures.

## Conclusion

This work examined the mechanical, morphological and thermal behavior of PE/PP blends and their modification with micro and nano fillers. The 75% HDPE blend showed the best balance among the unfilled compositions and was selected for further improvement. Micro-silica at 5 wt% enhanced all properties of the blend, increasing yield strength by 17%, elongation at break by 43%, and toughness by 72%, this improvement was also accompanied by an increase in Shore D hardness by 5%, indicating enhanced resistance to surface deformation, confirming its effective reinforcement role. Carbon black mainly increased the yield strength and hardness. In contrast, EPDM reduced performance across all metrics. The most remarkable improvements were achieved with nano-silica, even at low concentrations. At 1 wt%, nano-silica, the yield strength increased by 13%, elongation at break increased by 98%, and toughness by 120%. The 3 wt% nano-silica formulation delivered the best overall performance, with increasing in yield strength by 13%, elongation at break by 120%, toughness by 150%, compared with the neat blend. Although the 5 wt% nano-silica composition remained substantially stronger and tougher than the base blend, its marginal decline compared with the 3 wt% composition may result from early-stage filler aggregation. In terms of shore D hardness, all nano-silica blends exhibited a consistent increase to approximately 5%, comparable to that achieved with micro-silica. Thermogravimetric analysis confirmed that nano-silica demonstrated the most pronounced stabilizing effect. In summary, the results show that well-dispersed nano silica is the most efficient modifier for the PE/PP blend. These improved nanocomposites show performance suitable for many engineering and industrial application.

## Data Availability

The datasets used or analyzed during the study available from the corresponding author on reasonable request.
